# Modeling the seasonal epidemic of human brucellosis in China: A comparative time series analysis

**DOI:** 10.1371/journal.pone.0344908

**Published:** 2026-03-25

**Authors:** Yuqi Jiang, Jinhua Zhao, Jiang Long, Ping Deng, Shenglin Qin, Yang Zhang

**Affiliations:** 1 Department of Public Health, Qinghai University Medical College, Xining, Qinghai Province, China; 2 Department of Operations, Qinghai Center for Disease Control and Prevention, Xining, Qinghai Province, China; 3 Chongqing Municipal Academy of Preventive Medicine, Liangjiang New Area, Chongqing, China; Hamadan University of Medical Sciences, IRAN, ISLAMIC REPUBLIC OF

## Abstract

**Background:**

While time-series models have been applied to forecast brucellosis incidence in China, systematic comparisons of multiple models remain relatively limited. This study aimed to elucidate the epidemic characteristics of human brucellosis and to provide a comparative assessment of several time-series prediction models, in order to identify a suitable predictive framework for future incidence forecasting.

**Methods:**

Monthly and annual incidence rates (per 100,000 population) of brucellosis in China from January 2011 to December 2020 were used as raw data. Seven time-series models were developed and compared using R software (version 4.3.1): Seasonal Autoregressive Integrated Moving Average (SARIMA), Holt-Winters additive model, Holt-Winters multiplicative model, Neural Network Autoregressive (NNAR) model, Exponential Smoothing State Space (ETS) model, TBATS model, and Prophet model. A rolling-window cross-validation was applied to assess model stability. Model performance was evaluated using root mean square error (RMSE), mean absolute error (MAE), mean absolute percentage error (MAPE), and mean absolute scaled error (MASE).

**Results:**

Among the seven models evaluated, the Holt-Winters multiplicative model demonstrated the most stable and superior predictive performance on the test set (MAE = 0.034, RMSE = 0.040, MAPE = 14.881%, MASE = 0.891), which serves as strong evidence for its best generalization capability among the compared models.

**Conclusions:**

Given its stable and superior performance in the test set, the Holt-Winters multiplicative model is recommended for short-term brucellosis forecasting in China. It captures the characteristic spring-summer peak, and its integration into surveillance systems could enhance early warning and targeted interventions.

## 1. Introduction

Brucellosis is a zoonotic infectious disease caused by Brucella. It mainly affects domestic animals such as cattle, sheep, and pigs, and can also be transmitted to humans. It is also a Class B infectious disease specified in the Law of the People’s Republic of China on the Prevention and Control of Infectious Diseases [1–3]. Globally, the disease remains prevalent in regions including the Middle East, the Mediterranean, and parts of the Americas, with an estimated 16,000–20,000 human cases annually [4]. Osteoarticular involvement is the most common complication, usually manifesting as spondylitis, sacroiliitis, and peripheral arthritis, with great social harm [5,6]. The risk of brucellosis is increasing in environments with poverty and insufficient resources [7].

Accurate forecasting of brucellosis incidence is crucial for effective public health preparedness and resource allocation [8–11]. Strengthening dynamic monitoring and predictive capabilities is fundamental to reducing the disease burden in China [12]. While predictive modelling offers a pathway to improved early warning, existing research has predominantly relied on the Autoregressive Integrated Moving Average (ARIMA) model [13–15]. Although effective for linear trends and seasonality, the ARIMA framework may not fully capture complex nonlinear dynamics inherent in epidemiological time series data, potentially limiting forecasting accuracy and adaptability [16,17].

While time-series models have been applied to forecast brucellosis incidence in China, systematic comparisons of multiple models remain relatively limited [13,14]. To address this gap, this study contributes several key advancements. First, it systematically compares seven distinct models: Seasonal Autoregressive Integrated Moving Average (SARIMA), Holt‑Winters additive, Holt‑Winters multiplicative, Neural Network Autoregression (NNAR), Exponential Smoothing State Space (ETS), Trigonometric seasonality, Box-Cox transformation, ARMA errors, Trend and Seasonal components (TBATS), and Prophet model. Second, model performance is rigorously assessed using both rolling-window cross-validation on the training set and an independent test set to evaluate real‑world forecasting ability. Third, prediction accuracy is comprehensively measured with multiple error metrics, namely root mean square error (RMSE), mean absolute error (MAE), mean absolute percentage error (MAPE), and mean absolute scaled error (MASE), enabling a robust multi‑criterion evaluation. Through this approach, the study aims to identify the most reliable forecasting framework for brucellosis incidence in China.

## 2. Materials and methods

### 2.1. Ethics statement

In accordance with the Law of the People’s Republic of China on the Prevention and Control of Infectious Diseases, brucellosis is a statutory notifiable disease incorporated into the national public health surveillance system. The collection of anonymized, population-level surveillance data for such diseases is a mandated public health function and does not require separate ethical committee review. The data used in this study were obtained from the National Infectious Disease Surveillance Network. All data were anonymized and de-identified prior to analysis, and no personal privacy or human subject research was involved [18,19].

### 2.2. Data source

Monthly incidence rates of brucellosis (per 100,000 population) in China from January 2011 to December 2020 were obtained from the Chinese Public Health Science Center. This dataset consists of 120 consecutive months of population-standardized surveillance data, which were reviewed and aggregated by the Centers for Disease Control and Prevention. Data continuity was confirmed, with no missing months identified. To preserve the original epidemiological signal, no smoothing or outlier removal was applied. The complete series was partitioned into a training set (January 2011-December 2019) and an independent test set (January-December 2020) for model development and evaluation.

### 2.3. Data processing and analysis

The monthly brucellosis incidence data were programmatically collated and structured as a time series object in R (version 4.3.1) using the ‘ts’ function with a frequency of 12, spanning from January 2011 to December 2020. Data continuity was verified by comparing the sequence of months, confirming a complete series of 120 observations with no missing values; consequently, no imputation was required. To maintain the integrity of the raw epidemiological signal, no data smoothing or explicit outlier detection and removal was performed prior to model fitting.

All time-series modeling, forecasting, and evaluation were conducted within the R environment. The analysis leveraged a suite of specific packages and versions to ensure reproducibility: forecast (version 8.21) for fitting SARIMA, ETS, TBATS, and NNAR models as well as for generating predictions; tseries for conducting the Augmented Dickey-Fuller (ADF) and Ljung-Box statistical tests; prophet (version 1.0) for building the Prophet model; and supporting packages including tidyverse, tsibble, and urca for data manipulation and analysis.

A rolling-window cross-validation scheme was implemented on the training set to evaluate model stability. The configuration used a window length of 60 months, encompassing five full annual cycles to adequately capture long-term seasonal patterns. The forecast horizon was set to 6 months, and the window was rolled forward by a step of 3 months. This step size balanced computational efficiency with the need for sufficient overlapping windows to ensure a stable assessment. This process generated 15 distinct validation windows in total.

The optimal predictive model was defined as the one with the lowest combined performance on MAPE and RMSE while maintaining competitive scores on the complementary metrics. The brucellosis incidence data in this study featured a low and stable average level of 3.29 per 100,000 population with distinct annual seasonality, which supported the selection of MAPE and RMSE as dual primary metrics for a comprehensive performance evaluation.

### 2.4. SARIMA model

The SARIMA model is derived from the time series model proposed by British statistician Jenkins in the 1970s. It aims to determine the order of the model using autocorrelation and partial autocorrelation functions, enabling it to better handle time series data with seasonal patterns [20,21]. The strength of the SARIMA model lies in its ability to capture both seasonal and trend components of time series data. This allows it, through reasonable selection of model parameters, to be more suitable for accurate short-term and medium-term predictions [22].

In this study, the SARIMA model was implemented using the auto.arima() function from the forecast package (version 8.21) in R. The model orders (p, d, q) and seasonal orders (P, D, Q) were automatically selected through a comprehensive stepwise search procedure configured with stepwise = FALSE and approximation = FALSE. The selection aimed to minimize the Akaike Information Criterion (AIC), with the search space constrained to maximum non-seasonal orders of p = 3 and q = 3 and maximum seasonal orders of P = 2 and Q = 2. This parameter range is conventional for monthly time series and serves to mitigate overfitting while capturing essential dynamics. As shown in Equation (1):


$$\phi_{p}(B)\Phi_{P}(B^{s})(\text{1}-B{)}^{d}(\text{1}-B^{s}{)}^{D}x_{t}=\theta_{q}(B)\Theta_{Q}(B^{s})\varepsilon_{t}$$ (1)

Where ($B^{s}$) is the seasonal delay operator, and $\phi_{p}(B)$ and $\theta_{q}(B)$ are delay polynomial operators.

### 2.5. Holt−Winters model

This model combines components of trend, seasonality, and random error, integrating the characteristics of single exponential smoothing, double exponential smoothing, and triple exponential smoothing [23]. It can be divided into additive and multiplicative models. It assigns higher weights to recent observations in the time series, while the weights of distant observations decrease in a geometric progression. The selection of model parameters has a great impact on model performance, and methods such as cross-validation are usually needed to optimize these parameters.

In this study, both the additive and multiplicative Holt-Winters models were fitted using the HoltWinters() function in R. The smoothing parameters (alpha for level, beta for trend, and gamma for seasonality) were optimized by minimizing the squared one-step prediction error on the training set. For the final multiplicative model selected, the optimized parameters were alpha = 0.356, beta = 0.126, and gamma = 1.000.

### 2.6. NNAR model

The NNAR model can be regarded as a network composed of a series of neurons or nodes, which can describe and model complex nonlinear relationships and function forms [24,25]. If the network contains no hidden layers, the generated model will only have the characteristics of linear regression, showing a simple linear relationship.

In this study, the NNAR model was fitted using the nnetar() function from the forecast package. The network architecture was configured with 12 lagged inputs (p = 12) to capture annual patterns and 1 seasonal lag (P = 1) for seasonal adjustment. The number of neurons in the single hidden layer was set to 8, which was determined as optimal through a grid search aimed at balancing model capacity and the risk of overfitting. To ensure robust training, the model was fitted with 30 random repetitions to mitigate the effect of initial weight randomness, and the training process was limited to a maximum of 150 iterations. The specific formula of the model is expressed as (2):


$$y_{t}=f(y_{t-\text{1}},y_{t-\text{2}},...,y_{t-p};\theta )+\varepsilon_{t}$$ (2)

Where $y_{t}$ is the current value at time t; f is the neural network, which accepts historical observations and errors as inputs; $y_{t-\text{1}},y_{t-\text{2}},...,y_{t-p}$ are lagged input items of $y_{t};\;\theta$ is the parameter of the model; and $\varepsilon_{t}$ is the error term of the time series. The NNAR model can be regarded as a powerful prediction tool. It combines the flexibility of neural networks with the time series prediction ability of the ARIMA model, and adapts to different time series patterns by adjusting parameters adaptively.

### 2.7. ETS model

The ETS model is suitable for time series data with stable trends and seasonality, and can automatically adjust smoothing coefficients according to data characteristics [26]. The main parameters of the model include the smoothing coefficient, trend coefficient, and seasonal index. The seasonal index is used to control the impact of seasonality, and its value range in the model is usually between 0 and 1. During model construction, it is necessary to select the appropriate type of ETS model according to the trend and seasonal characteristics of the data.

In this study, the ETS model was implemented using the ets() function. The model’s error, trend, and seasonal components (ETS) were automatically selected from a range of possible combinations. The selection process was based on optimizing the corrected Akaike Information Criterion (AICc). The final fitted model was of type ETS (A, N, A), indicating an additive error, no trend, and additive seasonality structure.

### 2.8. TBATS model

The TBATS model innovatively introduces an exponential smoothing state space modeling framework. It is an advanced method in time series analysis, which can capture trends, seasonality, and periodicity in time series data simultaneously [27]. Based on seasonal decomposition and locally weighted regression, it can smooth and predict time series data, and can smooth seasonal components to reduce noise and random fluctuations [28].

In this study, the TBATS model was fitted using the tbats function. The model configuration incorporated a Box-Cox transformation, included both a trend component and a damped trend component, and accounted for ARMA errors in the residuals. The seasonal period was explicitly set to 12 months to match the frequency of the monthly incidence data.

### 2.9. Prophet model

The Prophet model is a modular additive regression model designed to forecast time series data with multiple seasonal patterns. It decomposes a time series into trend, seasonal, and holiday components, and is particularly robust to missing data and abrupt changes in trend [29–31]. The model uses a curve-fitting approach that allows for flexible adjustment of seasonal patterns and trend changepoints, making it suitable for datasets with pronounced and potentially changing periodicities.

In this study, the Prophet model was implemented using the prophet package version 1.0 in R. A linear growth trend was specified for the model. Multiplicative annual seasonality was enabled to better capture the observed pattern where seasonal fluctuations in brucellosis incidence scale with the overall level of the series. The flexibility of the seasonal component was controlled by setting the seasonality prior scale to 10. The trend flexibility was regulated by setting the changepoint prior scale to 0.05. The model was fitted on the training set and used to generate forecasts for the test period.

## 3. Results

### 3.1. Descriptive statistics

A total of 450,284 brucellosis cases were reported in China from 2011 to 2020, with 8 cumulative deaths and an average annual incidence rate of 3.29 per 100,000 population. Annual incidence rates were 2.85, 2.93, 3.21, 4.22, 4.18, 3.44, 2.79, 2.73, 3.15, and 3.37 per 100,000 population in sequence, with the lowest incidence observed in 2018. Our study demonstrated that the period from 2014 to 2015 was a peak. After that, it declined briefly but rose again in 2020, showing a trend of decreasing, then increasing, then decreasing, and subsequently resurging (Table 1).

**Table 1:** Incidence and mortality of brucellosis in China from 2011 to 2020.

Year	Number of cases	Incidence rate/100,000	Number of deaths	Mortality/100,000
2011	38,151	2.85	0	0.000,0
2012	39,515	2.93	1	0.000,1
2013	43,486	3.21	0	0.000,0
2014	57,222	4.22	2	0.000,1
2015	56,989	4.18	1	0.000,1
2016	47,139	3.44	2	0.000,1
2017	38,554	2.79	1	0.000,1
2018	37,947	2.73	0	0.000,0
2019	44,036	3.15	1	0.000,1
2020	47,245	3.37	0	0.000,0

### 3.2. Specific distribution

Between 2011 and 2020, the distribution of human brucellosis cases in China exhibited substantial spatial heterogeneity across provinces. During this period, multiple provinces reported cases, with particularly high disease burdens observed in Inner Mongolia Autonomous Region, Xinjiang Uygur Autonomous Region, Ningxia Hui Autonomous Region, Shanxi Province, and Heilongjiang Province. The average annual incidence rates in these regions reached 43.23, 21.11, 25.82, 13.95, and 13.69 per 100,000 population, respectively. Hebei Province, Gansu Province, and Qinghai Province were also identified as relatively high-risk areas, each reporting incidence rates exceeding 5 per 100,000 population (Table 2).

**Table 2:** Provincial distribution of Brucella in China from 2011 to 2020.

Province	Total number of cases	Annual average incidence rate/100,000
Beijing	1,216	0.57
Tianjin	1,543	1.04
Hebei	39,991	5.42
Shanxi	50,799	13.95
Inner Mongolia	108,370	43.23
Liaoning	21,669	4.95
Jilin	15,564	5.68
Heilongjiang	52,245	13.69
Shanghai	45	0.02
Jiangsu	1,038	0.13
Zhejiang	1,005	0.18
Anhui	957	0.15
Fujian	752	0.19
Jiangxi	436	0.09
Shandong	23,507	2.38
Henan	30,932	3.26
Hubei	1,277	0.22
Hunan	1,072	0.16
Guangdong	2,859	0.26
Guangxi	971	0.20
Hainan	83	0.09
Chongqing	270	0.09
Sichuan	581	0.07
Guizhou	506	0.14
Yunnan	1,877	0.39
Tibet	150	0.44
Shaanxi	9,173	2.42
Gansu	13,922	5.33
Qinghai	723	1.22
Ningxia	17,324	25.82
Xinjiang	49,427	21.11

Regarding age distribution, the majority of brucellosis cases occurred among young and middle-aged adults. The incidence rate demonstrated an initial increase followed by a decline with advancing age. The most affected demographic was the 40–64 age group, with the highest number of cases reported in the 45–49 subgroup (65,796 cases), followed by the 50–54 subgroup (64,305 cases). In terms of incidence, the 50–54 age group exhibited the highest average annual rate at 7.46 per 100,000, followed by the 55–59 group (5.96 per 100,000) and the 60–64 group (6.40 per 100,000). These findings indicate that middle-aged adults represent the population at greatest risk for brucellosis infection (Table 3).

**Table 3:** Age distribution of Brucella in China from 2011 to 2020.

Age	Total number of cases	Annual average incidence rate/100,000
0-	186	0.12
1-	592	0.35
2-	922	0.55
3-	966	0.62
4-	788	0.53
5-	806	0.56
6-	769	0.55
7-	703	0.42
8-	702	0.37
9-	759	0.50
10-	4,129	0.60
15-	5,928	0.70
20-	13,792	1.15
25-	26,014	2.46
30-	33,117	3.53
35-	39,085	3.63
40-	53,517	4.59
45-	65,796	5.27
50-	64,305	7.46
55-	52,432	5.96
60-	43,412	6.40
65-	25,034	4.96
70-	10,502	2.81
75-	4,221	1.54
80-	1,408	0.86
≥85	397	0.45

### 3.3. Trend analysis and seasonal decomposition

**Fig 1:**
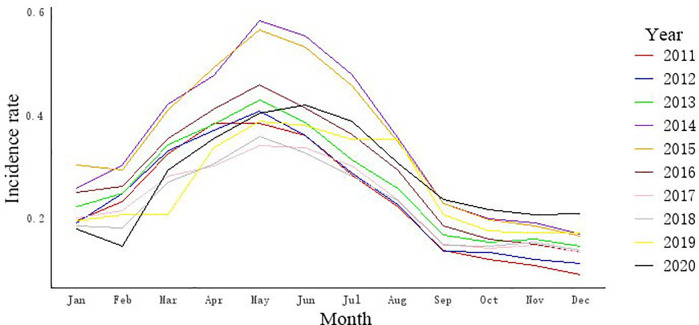
Monthly distribution changes of brucellosis in China from 2011 to 2020.

**Fig 2:**
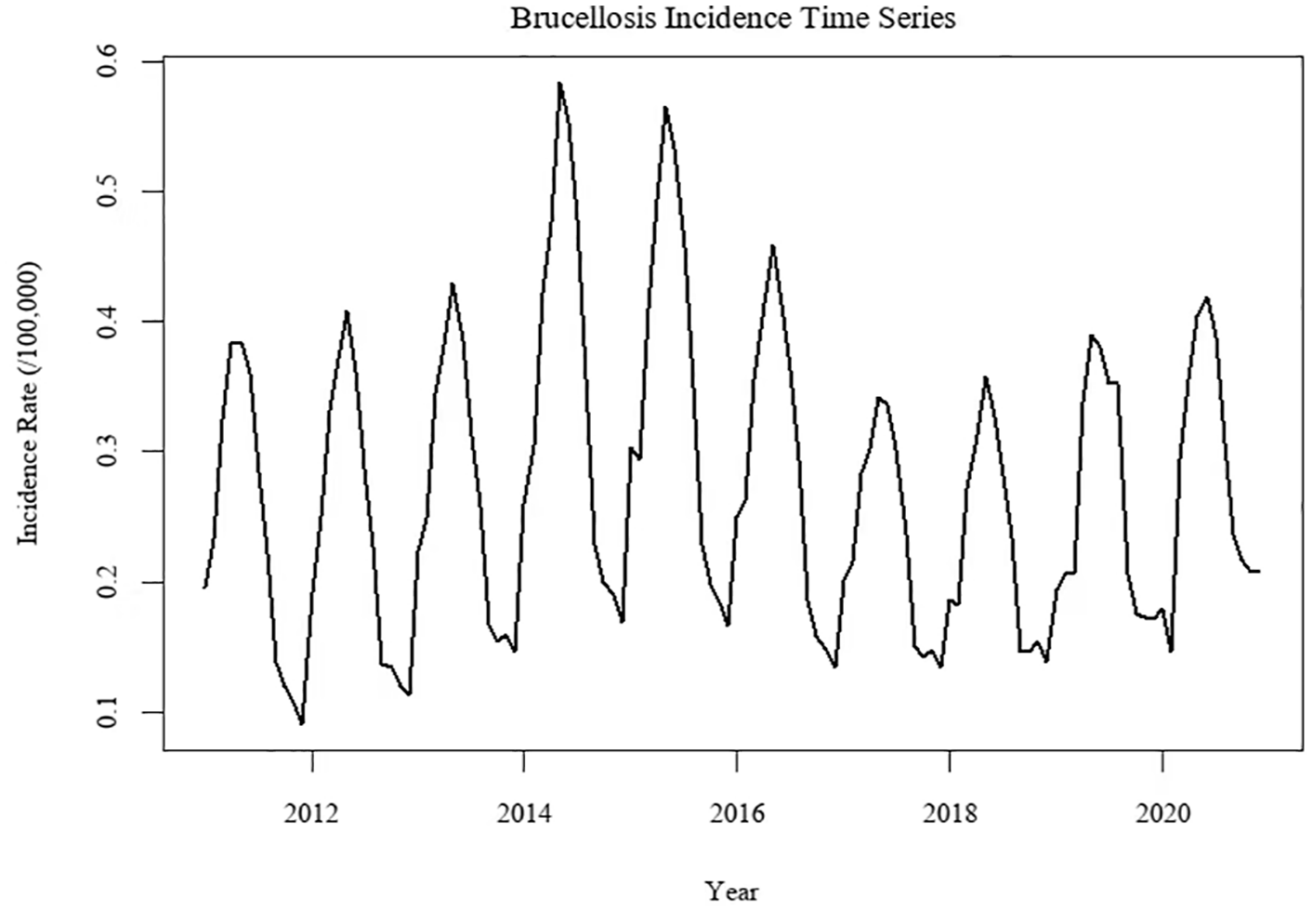
Time series distribution of brucellosis in China from 2011 to 2020.

**Fig 3:**
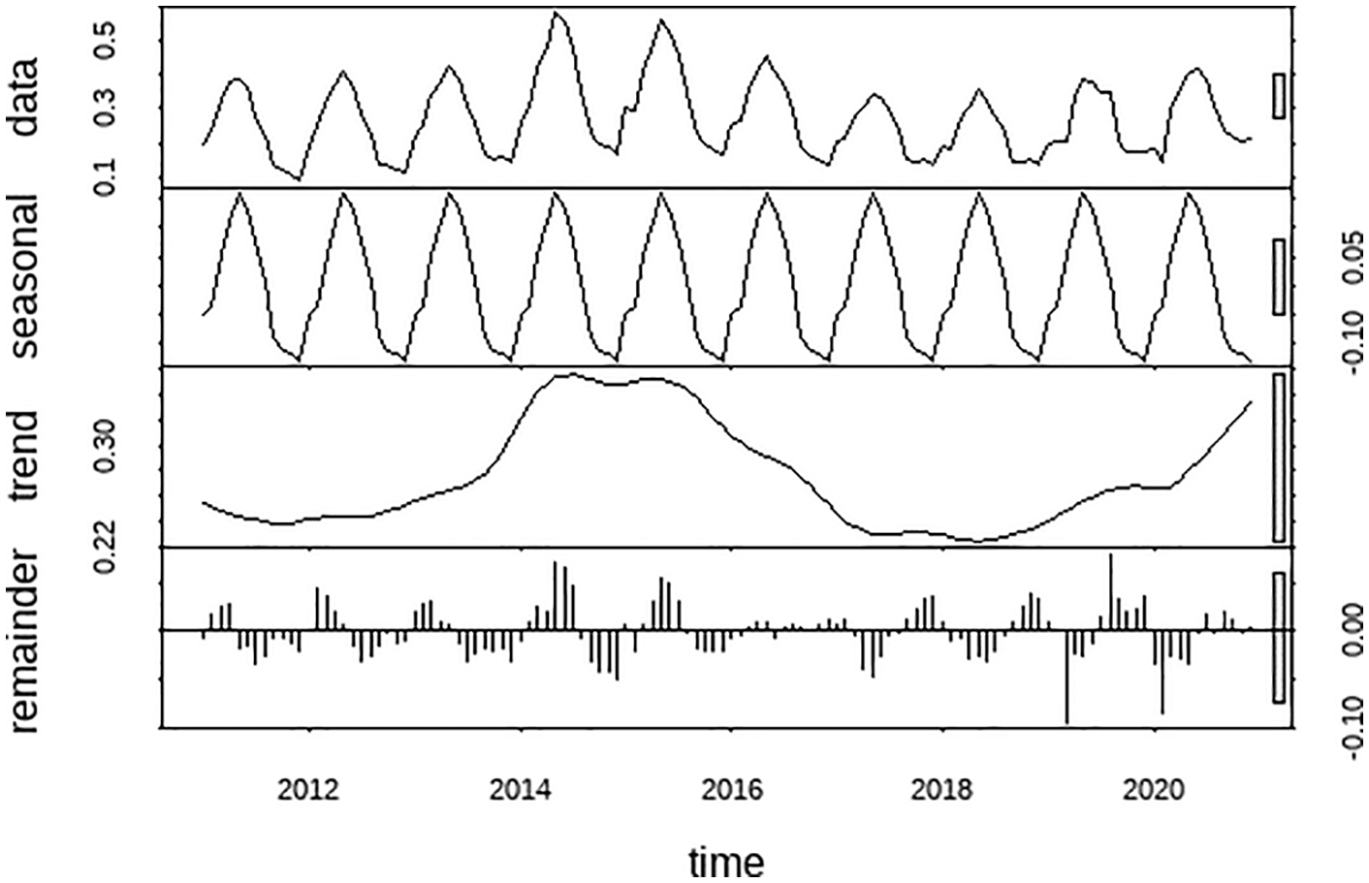
Seasonal Trend Decomposition of Brucellosis in China from 2011 to 2020.

The monthly incidence time series of brucellosis in China from January 2011 to December 2020 is presented in Figs 1 and 2. The series exhibited clear annual fluctuations, with a consistent seasonal peak occurring between April and June each year. Statistical tests confirmed the characteristics of the series: the ADF test indicated stationarity (*p* < 0.05), while the Ljung-Box test rejected the null hypothesis of white noise (*p* < 0.001), confirming the presence of serial correlation suitable for modeling Fig 3.

**Fig 4:**
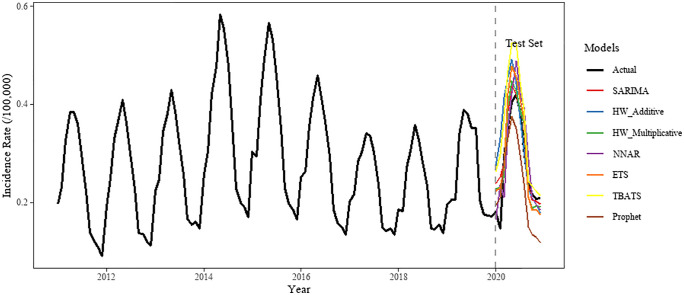
Results of five prediction models for brucellosis in China from 2011 to 2020.

Seasonal-Trend decomposition using LOESS (STL) was applied to decompose the original series into trend, seasonal, and remainder components (Fig 4). The decomposition revealed that the incidence rate peaked around 2015, followed by a general declining trend, with a notable resurgence observed in 2020. The seasonal component clearly captured the recurring spring-summer epidemic pattern, providing a distinct seasonal signal for subsequent forecasting models.

### 3.4. Model performance evaluation

On the training set, the lowest error metrics were recorded by the NNAR model, with MAE, RMSE, and MAPE values of 0.001, 0.001, and 0.435%, respectively. Comparatively low error values were also observed for the TBATS, SARIMA, and ETS models, indicating a good fit to the training data (Fig 4 and Table 4).

**Table 4:** Indicator Results of the Chinese Brucellosis Prediction Model from 2011 to 2020.

Data set	Model	MAE	RMSE	MASE	MAPE(%)
Training set	SARIMA	0.015	0.023	0.392	5.687
	Holt-Winters additive	0.044	0.060	1.160	16.532
	Holt-Winters multiplication	0.041	0.054	1.088	15.760
	NNAR	0.001	0.001	0.021	0.435
	ETS	0.018	0.025	0.462	6.468
	TBATS	0.016	0.021	0.420	6.093
	Prophet	0.026	0.033	0.678	10.253
Test set	SARIMA	0.028	0.041	0.745	13.476
	Holt-Winters additive	0.057	0.080	1.506	25.516
	Holt-Winters multiplication	0.034	0.040	0.891	14.881
	NNAR	0.041	0.052	1.073	16.430
	ETS	0.044	0.049	1.154	17.713
	TBATS	0.070	0.084	1.843	27.065
	Prophet	0.059	0.066	1.544	23.667

**Fig 5:**
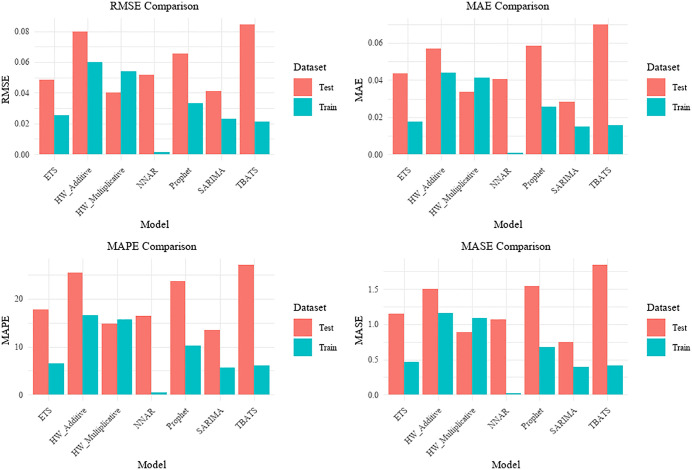
Comparison of Model Evaluation.

On the independent test set (year 2020), the Holt-Winters multiplicative model demonstrated strong predictive performance, achieving the lowest RMSE (0.040) and MAE (0.034), with a MAPE of 14.88%. Although the SARIMA model yielded a slightly lower MAPE (13.48%), its higher RMSE (0.041) suggested less stability in handling extreme values. A notable discrepancy was identified for the NNAR model, whose MAPE increased substantially from 0.435% on the training set to 16.430% on the test set, suggesting a risk of overfitting. In contrast, higher test-set MAPE values were observed for the TBATS model (27.065%) and the Holt-Winters additive model (25.516%) (Fig 5).

### 3.5. Rolling-window cross-validation results

The cross-validation results indicated that the TBATS model achieved the lowest average MAPE (14.56%), followed by Prophet (15.49%) and ETS (15.97%). In this internal validation, the Holt-Winters multiplicative model yielded a higher average MAPE of 20.43%. This apparent discrepancy is reconciled by examining performance on the completely unseen test set. As shown in Section 3.4, the Holt-Winters multiplicative model attained the lowest MAPE (14.88%) on the independent 2020 data, demonstrating superior generalization.

This contrast suggests a key difference in model behaviour. Flexible models like TBATS may have adapted closely to specific local patterns and noise within the historical rolling windows of the validation, leading to superior in-sample scores. In contrast, the Holt-Winters multiplicative model, with its parsimonious multiplicative seasonal structure, likely captured a more fundamental and stable seasonal trend. While this resulted in a slightly poorer fit to certain historical sub-periods during cross-validation, it proved to be a more robust and transferable pattern for forecasting the truly unseen data of 2020, leading to more reliable out-of-sample predictions (Table 5).

**Table 5:** Model Cross-Validation Evaluation Indicators.

Data set	Model	MAE	RMSE	MASE	MAPE(%)
Cross-Validation	SARIMA	0.049	0.054	1.192	19.941
	Holt-Winters additive	0.070	0.076	1.668	32.253
	Holt-Winters multiplication	0.053	0.060	1.308	20.431
	NNAR	0.049	0.057	1.183	18.175
	ETS	0.044	0.049	1.126	15.968
	TBATS	0.038	0.043	0.937	14.558
	Prophet	0.040	0.043	1.001	15.490

### 3.6. Model prediction for 2021

Based on its superior and stable performance on the test set, the Holt-Winters multiplicative model was selected to generate a forecast for the year 2021. The model was applied to the complete time series, concluding in December 2020. The resulting forecast for the 12 months of 2021 indicated a unimodal distribution for the monthly incidence rate, with a peak predicted during the spring-summer period. The specific forecasted values are presented in Table 6. This application demonstrates the model’s utility for prospective, short-term epidemiological forecasting.

**Table 6:** Model’s forecast results for 2021.

Month	1	2	3	4	5	6	7	8	9	10	11	12
Predictive value	0.23	0.21	0.36	0.45	0.52	0.52	0.47	0.39	0.27	0.23	0.22	0.21

### 3.7. Residual analysis of model diagnosis

A comprehensive diagnostic analysis of the Holt-Winters multiplicative seasonal model’s residuals was conducted to evaluate its specification. The statistical metrics revealed a residual mean near zero (−0.002) and a standard deviation of 0.029, with a kurtosis of 4.108 indicating a slightly leptokurtic distribution. The Ljung-Box test (*p* < 0.000) and the Shapiro-Wilk test (*p* < 0.001) both rejected their null hypotheses, suggesting the presence of residual autocorrelation and a deviation from normality. Importantly, the identified autocorrelation is primarily linked to a subtle, unmodeled seasonal component, as the model demonstrably captured the dominant trend and core seasonal patterns with high accuracy, evidenced by its superior performance on the test set.

**Fig 6:**
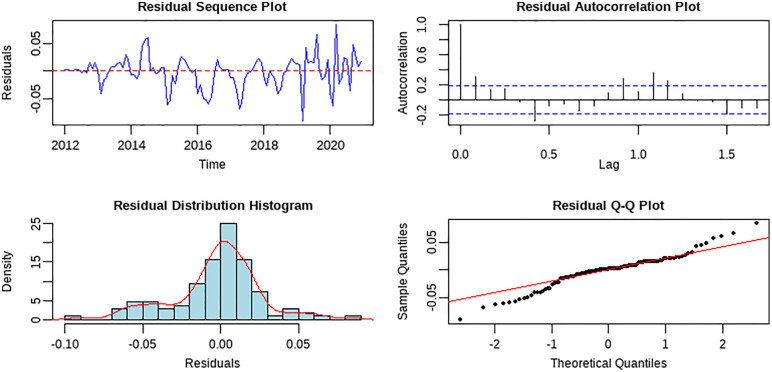
Residual diagnostic plots for the Holt-Winters multiplicative seasonal forecasting model.

Visual inspection of the residuals, as shown in Fig 6, supports this interpretation. While the sequence plot shows fluctuations around zero without a clear trend, the autocorrelation function plot reveals a significant peak at the seasonal lag of 12 (approximately 0.4). This confirms the persistence of a minor seasonal signal within the residuals. It is noteworthy, however, that the amplitude of this residual seasonality is small relative to the model’s fitted values. This implies that the Holt-Winters model successfully captured the primary, deterministic seasonal structure of the brucellosis incidence data, with the residual autocorrelation representing a secondary, less pronounced pattern. Overall, the model demonstrates a robust and effective decomposition of the time series into its fundamental components.

## 4. Discussion

Brucellosis remains a significant public health concern in China, associated with a considerable disease burden as evidenced by the reported cases from 2011 to 2020 [32–34]. The observed incidence pattern, characterized by a distinct unimodal seasonal peak from April to June, aligns with findings from other regions and is consistent with known epidemiological drivers [35–39]. This seasonal surge is likely attributable to heightened livestock reproductive activities and consequent occupational exposure during spring and summer, coupled with environmental conditions that favor pathogen survival [40,41]. It is necessary to increase disease prevention measures to reduce healthcare-related burdens [42]. The resurgence of cases in 2020 highlights the persistent and dynamic nature of the epidemic, necessitating ongoing vigilance.

A primary contribution of this study lies in the systematic comparison of seven time-series forecasting models. The analysis revealed that model performance on the training set was not a reliable indicator of true predictive capability. For instance, the NNAR model achieved a near-perfect in-sample fit but exhibited a marked performance decline on the independent test set, a clear signature of overfitting. This suggests that its complex, nonlinear structure may have adapted too closely to idiosyncratic patterns or noise within the specific training period, compromising its ability to generalize [43–45]. Similarly, advanced models like TBATS and Prophet, despite their theoretical strengths in handling complex seasonality, did not achieve superior out-of-sample accuracy in this application, indicating potential over-parameterization or a mismatch with the fundamental structure of the incidence series.

Conversely, the Holt-Winters multiplicative model demonstrated robust and superior predictive accuracy on the test set. Its stability was further corroborated by its performance in the rolling-window cross-validation. The effectiveness of this model can be attributed to its structural congruence with the key characteristic of the brucellosis incidence data: a seasonal amplitude that scales multiplicatively with the local level of the series. This feature allows it to accurately capture the observed phenomenon where the absolute size of the annual spring-summer peak varies in proportion to the underlying trend, a relationship that additive seasonal models cannot represent adequately [46–48]. Thus, its optimal performance stems from a parsimonious design that correctly specifies the interaction between trend and seasonality inherent in the data.

Several limitations of this study should be acknowledged. The models were developed using historical incidence data alone, without incorporating potential external predictors such as climatic variables, livestock population data, or detailed intervention records, which might improve forecast precision. Secondly, while cross-validation was employed, the models are inherently more suited to short-term forecasting given the stable seasonal patterns in the near term. Future research could explore integrated models that combine time-series approaches with explanatory variables. Thirdly, the analysis was constrained to nationally aggregated monthly data up to 2020. Future studies could benefit from incorporating more recent data, higher-frequency (e.g., weekly) reporting, and spatially explicit modeling at the prefecture or county level to capture local heterogeneities. Finally, although the data originated from the national surveillance system, which is authoritative, the possibility of underreporting or misclassification, common in passive surveillance, cannot be entirely excluded.

In conclusion, through a comparative evaluation, the Holt-Winters multiplicative model was identified as the most accurate and stable choice for short-term forecasting of brucellosis incidence in China. Its implementation within surveillance frameworks could enhance early warning capabilities, supporting timely and targeted public health actions before the seasonal high-risk period.

## 5. Conclusion

This study confirms a distinct spring-summer seasonal peak in human brucellosis incidence in China, with middle-aged adults constituting the primary affected population. Among seven time-series models compared, the Holt-Winters multiplicative model demonstrated the most stable and accurate short-term forecasts. Its superior performance stems from its multiplicative seasonal structure, which effectively captures the key epidemiological pattern wherein seasonal fluctuation amplitudes scale proportionally with the underlying trend level. Integrating this robust model into the national surveillance framework would enable proactive early warnings and support targeted interventions prior to the annual high-risk period, thereby strengthening brucellosis control strategies in China.

## Supporting information

S1 File The original data of the article. (XLSX)

S2 File The original code of the article. (PDF)
