# Supplementary material for: Modeling the seasonal epidemic of human brucellosis in China: A comparative time series analysis
Source: PLoS One. 2026 Mar 25;21(3):e0344908. doi: 10.1371/journal.pone.0344908 (PMC13016291; doi:10.1371/journal.pone.0344908)
Supplement: S2 File — (PDF) [file pone.0344908.s002.pdf]

# The codes used in the data

```
#
==========================================================================
======
    # Brucellosis Incidence Rate Time Series Forecasting - Comprehensive Model Comparison
    # Version: 2.1 (Optimized and Fixed Logical Errors)
    #
==========================================================================
======

    # 1. Clear Environment and Load Packages
============================================
    cat("=== Brucellosis Incidence Rate Forecasting Analysis ===\n")
    cat("Start time:", format(Sys.time(), "%Y-%m-%d %H:%M:%S"), "\n\n")

    # Clear environment
    rm(list = ls())
    gc()

    # Load required packages
    required_packages <- c("readxl", "tidyverse", "forecast", "tseries",
                           "purrr", "urca", "tsibble", "ggplot2",
                           "lubridate", "prophet", "parallel", "doParallel",
                           "gridExtra", "scales", "showtext", "dplyr")

    cat("1. Checking and loading required R packages...\n")
    for (pkg in required_packages) {
      if (!require(pkg, character.only = TRUE)) {
        install.packages(pkg, dependencies = TRUE)
        library(pkg, character.only = TRUE)
      }
    }

    # Load Times New Roman font
    if ("showtext" %in% installed.packages()[, "Package"]) {
      showtext::showtext_auto()
      font_add("Times New Roman", "times.ttf")
      base_font <- "Times New Roman"
    } else {
      base_font <- "serif"   # Fallback to serif font
    }

    # 2. Custom Functions
```

================================================================

```r
cat("2. Defining custom functions...\n")

# Calculate MASE function (considering seasonality)
calculate_mase <- function(actual, predicted, train_data, frequency = 12) {
  naive_error <- mean(abs(diff(train_data, lag = frequency)), na.rm = TRUE)
  if (naive_error == 0) {
    return(NA)
  }
  mean_abs_error <- mean(abs(actual - predicted), na.rm = TRUE)
  return(mean_abs_error / naive_error)
}

# Unified accuracy calculation function
calculate_accuracy <- function(actual, predicted, train_series) {
  actual_numeric <- as.numeric(actual)
  predicted_numeric <- as.numeric(predicted)

  # Filter out NA values
  valid_idx <- !is.na(actual_numeric) & !is.na(predicted_numeric) & actual_numeric > 0
  actual_valid <- actual_numeric[valid_idx]
  predicted_valid <- predicted_numeric[valid_idx]

  if (length(actual_valid) == 0) {
    return(c(RMSE = NA, MAE = NA, MAPE = NA, MASE = NA))
  }

  mse <- mean((actual_valid - predicted_valid)^2, na.rm = TRUE)
  mae <- mean(abs(actual_valid - predicted_valid), na.rm = TRUE)
  mape <- mean(abs((actual_valid - predicted_valid) / actual_valid) * 100, na.rm = TRUE)
  mase <- calculate_mase(actual_valid, predicted_valid, train_series)

  return(c(RMSE = sqrt(mse), MAE = mae, MAPE = mape, MASE = mase))
}

# Rolling window cross-validation function (optimized version)
fast_rolling_window_cv <- function(model_func, data,
                                   h = 6,
                                   window_size = 60,
                                   step_size = 3,
                                   progress = TRUE) {
  n <- length(data)
  if (n <= window_size + h) {
    stop("Data length insufficient for specified cross-validation")
```

```
    }

    # Determine number of windows
    start_indices <- seq(1, n - window_size - h + 1, by = step_size)
    n_windows <- length(start_indices)

    if (progress) {
      cat(sprintf("    Rolling window cross-validation: %d windows\n", n_windows))
      pb <- txtProgressBar(min = 0, max = n_windows, style = 3, char = "=")
    }

    # Store performance for each window
    window_performance <- list()
    window_errors <- list()

    for (i in seq_along(start_indices)) {
      start_idx <- start_indices[i]
      end_idx <- start_idx + window_size - 1

      train_window <- window(data, start = time(data)[start_idx],
                                      end = time(data)[end_idx])
      test_window <- window(data, start = time(data)[end_idx + 1],
                                     end = time(data)[end_idx + h])

      # Train model
      tryCatch({
        model <- model_func(train_window)

        # Forecast
        if (inherits(model, "nnetar")) {
          fc <- forecast(model, h = h, PI = FALSE)
          predicted <- as.numeric(fc$mean)
        } else if (inherits(model, "prophet")) {
          # Fixed logical error: Use the trained Prophet model from model_func instead of
retraining
          # Get training window date range
          start_time <- time(train_window)[1]
          start_year <- floor(start_time)
          start_month <- round((start_time - start_year) * 12) + 1
          start_date <- as.Date(paste(start_year, start_month, "01", sep = "-"))
          # Forecast dates
          future_dates <- seq.Date(max(seq.Date(start_date, by = "month", length.out =
length(train_window))) + months(1),
                                   by = "month", length.out = h)
```

```
            future_df <- data.frame(ds = future_dates)
            fc_pred <- predict(model, future_df)
            predicted <- fc_pred$yhat
          } else {
            fc <- forecast(model, h = h, level = FALSE)
            predicted <- as.numeric(fc$mean)
          }

          # Calculate performance metrics
          actual <- as.numeric(test_window)
          perf <- calculate_accuracy(actual, predicted, as.numeric(train_window))

          window_performance[[i]] <- perf
        }, error = function(e) {
          window_performance[[i]] <- c(RMSE = NA, MAE = NA, MAPE = NA, MASE =
NA)
          window_errors[[i]] <- paste("Window", i, "error:", e$message)
        })

        if (progress) {
          setTxtProgressBar(pb, i)
        }
      }

      if (progress) {
        close(pb)
      }

      # Print any errors that occurred
      if (length(window_errors) > 0) {
        cat("    Errors occurred in some windows:\n")
        for (err in window_errors) {
          cat("    -", err, "\n")
        }
      }

      # Calculate average performance (excluding NA values)
      perf_matrix <- do.call(rbind, window_performance)
      avg_performance <- colMeans(perf_matrix, na.rm = TRUE)

      return(list(
        avg_performance = avg_performance,
        window_performance = window_performance,    # Keep window-level performance
data
```

```
      n_windows = n_windows,
      errors = window_errors
    ))
  }

  # Unified model evaluation function
  evaluate_model <- function(model, train_data, test_data, model_type) {
    # Get predictions
    if (model_type == "Prophet") {
      # Fixed logical error: Remove hardcoded dates, dynamically generate train/test set dates
      train_start_date <- as.Date(paste(start(train_data)[1], start(train_data)[2], "01", sep = "-
"))
      train_future <- data.frame(ds = seq.Date(train_start_date, by = "month", length.out =
length(train_data)))
      train_pred <- predict(model, train_future)$yhat

      test_start_date <- as.Date(paste(start(test_data)[1], start(test_data)[2], "01", sep = "-"))
      test_future <- data.frame(ds = seq.Date(test_start_date, by = "month", length.out =
length(test_data)))
      test_pred <- predict(model, test_future)$yhat
    } else if (model_type == "NNAR") {
      # NNAR model
      train_pred <- as.numeric(fitted(model))
      fc <- forecast(model, h = length(test_data), PI = FALSE)
      test_pred <- as.numeric(fc$mean)
    } else if (model_type %in% c("HW_Additive", "HW_Multiplicative")) {
      # Holt-Winters models
      train_pred <- as.numeric(fitted(model))
      fc <- forecast(model, h = length(test_data))
      test_pred <- as.numeric(fc$mean)
    } else {
      # SARIMA, ETS, TBATS
      train_pred <- as.numeric(fitted(model))
      fc <- forecast(model, h = length(test_data))
      test_pred <- as.numeric(fc$mean)
    }

    # Calculate accuracies
    train_acc <- calculate_accuracy(as.numeric(train_data), train_pred, as.numeric(train_data))
    test_acc <- calculate_accuracy(as.numeric(test_data), test_pred, as.numeric(train_data))

    return(list(
      train_accuracy = train_acc,
      test_accuracy = test_acc,
```

```
        train_predictions = train_pred,
        test_predictions = test_pred
    ))
}

# 3. Data Loading and Preprocessing
================================================
cat("\n3. Data loading and preprocessing...\n")

# Fixed logical error: Add file selection error tolerance to avoid direct error from hardcoded
path
cat("    Please select the data file (Excel format)...\n")
file_path <- tryCatch({
    choose.file(default = "C:/Users/jj/Desktop/Dataset.xlsx",
                caption = "Select Brucellosis Data File")
}, error = function(e) {
    "C:/Users/jj/Desktop/Dataset.xlsx"
})

# Import data
d1 <- read_xlsx(file_path)

# Check data structure
cat("    Data structure:\n")
str(d1)
cat("    First few rows:\n")
print(head(d1))
cat("    Last few rows:\n")
print(tail(d1))

# Create time series
ts_d1 <- ts(d1$y, frequency = 12, start = c(2011, 1), end = c(2020, 12))

# Check data continuity
cat("    Checking data continuity...\n")
all_months <- seq.Date(as.Date("2011-01-01"), as.Date("2020-12-01"), by = "month")
actual_months <- as.Date(paste(floor(time(ts_d1)),
                               round((time(ts_d1) - floor(time(ts_d1))) * 12) + 1,
                               "01", sep = "-"))
if (length(actual_months) != length(all_months)) {
    warning("Data may have missing months! Expected ", length(all_months),
            " months, got ", length(actual_months), " months.")
} else {
    cat("    Data is continuous, all months present.\n")
```

```
}

cat(sprintf("    Data overview: %d months (%.0f years)\n", length(ts_d1), length(ts_d1)/12))
cat(sprintf("    Time range: %.0f/%.0f to %.0f/%.0f\n",
                      start(ts_d1)[1], start(ts_d1)[2],
                      end(ts_d1)[1], end(ts_d1)[2]))
cat(sprintf("    Mean: %.3f, SD: %.3f, Min: %.3f, Max: %.3f\n",
                      mean(ts_d1, na.rm = TRUE), sd(ts_d1, na.rm = TRUE),
                      min(ts_d1, na.rm = TRUE), max(ts_d1, na.rm = TRUE)))

# Check for missing values
if (any(is.na(ts_d1))) {
   warning("Time series contains missing values at positions: ",
                paste(which(is.na(ts_d1)), collapse = ", "))
   # Impute missing values using linear interpolation
   ts_d1 <- na.approx(ts_d1)
   cat("    Missing values imputed using linear interpolation.\n")
}

# Split into training and test sets
train_ts <- window(ts_d1, end = c(2019, 12))    # 2011-2019
test_ts <- window(ts_d1, start = c(2020, 1))    # 2020

cat(sprintf("    Training set: %d months (%.1f years)\n", length(train_ts), length(train_ts)/12))
cat(sprintf("    Test set: %d months (Year 2020)\n", length(test_ts)))

# 4. Basic Exploratory Analysis
=====================================================
cat("\n4. Basic exploratory analysis...\n")

# 4.1 Time series plot
par(mfrow = c(1, 2), mar = c(4, 4, 2, 1))
plot(ts_d1, main = "Brucellosis Incidence Time Series",
        xlab = "Year", ylab = "Incidence Rate (/100,000)", lwd = 2,
        family = base_font)

# 4.2 Seasonal decomposition
decomposed <- stl(ts_d1, s.window = "periodic")
plot(decomposed, main = "Seasonal-Trend Decomposition",
        family = base_font)

# 4.3 Unit root test
adf_result <- adf.test(ts_d1)
cat(sprintf("    ADF test: p-value = %.4f, %s\n",
```

```
                    adf_result$p.value,
                    ifelse(adf_result$p.value < 0.05, "Stationary series", "Non-stationary series")))

    # 4.4 White noise test
    ljung_result <- Box.test(ts_d1, type = "Ljung-Box")
    cat(sprintf("    Ljung-Box test: p-value = %.4f, %s\n",
                    ljung_result$p.value,
                    ifelse(ljung_result$p.value < 0.05, "Non-white noise series", "White noise
series")))

    # Reset graphical parameters
    par(mfrow = c(1, 1))

    # 5. Model Training and Cross-Validation
=============================================
    cat("\n5. Model training and cross-validation...\n")

    # Initialize storage objects
    models <- list()
    train_accuracies <- list()
    test_accuracies <- list()
    cv_results <- list()

    # Cross-validation settings
    cv_window_size <- 60    # 5 years
    cv_h <- 6                   # Forecast 6 months
    cv_step <- 3              # Step of 3 months
    cat(sprintf("    Cross-validation settings: window size=%d months, forecast horizon=%d
months, step size=%d months\n",
                    cv_window_size, cv_h, cv_step))

    # Set random seed for reproducibility
    set.seed(2020)

    # 5.1 SARIMA model
    cat("\n5.1 SARIMA model training...\n")
    fit_sarima <- auto.arima(train_ts,
                                  d = NA, D = NA,
                                  max.p = 3, max.q = 3,
                                  max.P = 2, max.Q = 2,
                                  seasonal = TRUE,
                                  ic = "aic",
                                  stepwise = FALSE,
                                  approximation = FALSE,
```

```
                              trace = FALSE)

    cat("    Performing SARIMA cross-validation...\n")
    sarima_cv_func <- function(data) {
      auto.arima(data,
                 d = NA, D = NA,
                 max.p = 3, max.q = 3,
                 max.P = 2, max.Q = 2,
                 seasonal = TRUE,
                 ic = "aic",
                 stepwise = FALSE,
                 approximation = FALSE,
                 trace = FALSE)
    }

    cv_sarima <- fast_rolling_window_cv(sarima_cv_func, train_ts,
                                        h = cv_h, window_size = cv_window_size,
                                        step_size = cv_step)

    cv_results$SARIMA <- cv_sarima$avg_performance
    cat(sprintf("    Cross-validation completed, %d windows\n", cv_sarima$n_windows))

    # Evaluate SARIMA model
    sarima_eval <- evaluate_model(fit_sarima, train_ts, test_ts, "SARIMA")
    models$SARIMA <- fit_sarima
    train_accuracies$SARIMA <- sarima_eval$train_accuracy
    test_accuracies$SARIMA <- sarima_eval$test_accuracy

    cat(sprintf("    Model type: %s\n", fit_sarima$arma))
    cat(sprintf("    Training set - RMSE: %.4f, MAE: %.4f, MAPE: %.3f%%, MASE: %.3f\n",
                sarima_eval$train_accuracy["RMSE"], sarima_eval$train_accuracy["MAE"],
                sarima_eval$train_accuracy["MAPE"],
sarima_eval$train_accuracy["MASE"]))
    cat(sprintf("    Test set - RMSE: %.4f, MAE: %.4f, MAPE: %.3f%%, MASE: %.3f\n",
                sarima_eval$test_accuracy["RMSE"], sarima_eval$test_accuracy["MAE"],
                sarima_eval$test_accuracy["MAPE"], sarima_eval$test_accuracy["MASE"]))
    cat(sprintf("    Cross-validation - RMSE: %.4f, MAE: %.4f, MAPE: %.3f%%,
MASE: %.3f\n",
                cv_results$SARIMA["RMSE"], cv_results$SARIMA["MAE"],
                cv_results$SARIMA["MAPE"], cv_results$SARIMA["MASE"]))

    # 5.2 Holt-Winters additive model
    cat("\n5.2 Holt-Winters additive model training...\n")
    fit_hw_add <- HoltWinters(train_ts, seasonal = "additive")
```

```
cat("    Performing Holt-Winters additive cross-validation...\n")
hw_add_cv_func <- function(data) {
  HoltWinters(data, seasonal = "additive")
}

cv_hw_add <- fast_rolling_window_cv(hw_add_cv_func, train_ts,
                                    h = cv_h, window_size = cv_window_size,
                                    step_size = cv_step)

cv_results$HW_Additive <- cv_hw_add$avg_performance
cat(sprintf("    Cross-validation completed, %d windows\n", cv_hw_add$n_windows))

# Evaluate HW additive model
hw_add_eval <- evaluate_model(fit_hw_add, train_ts, test_ts, "HW_Additive")
models$HW_Additive <- fit_hw_add
train_accuracies$HW_Additive <- hw_add_eval$train_accuracy
test_accuracies$HW_Additive <- hw_add_eval$test_accuracy

cat(sprintf("    Model parameters: alpha=%.3f, beta=%.3f, gamma=%.3f\n",
            fit_hw_add$alpha, fit_hw_add$beta, fit_hw_add$gamma))
cat(sprintf("    Training set - RMSE: %.4f, MAE: %.4f, MAPE: %.3f%%, MASE: %.3f\n",
            hw_add_eval$train_accuracy["RMSE"],
hw_add_eval$train_accuracy["MAE"],
            hw_add_eval$train_accuracy["MAPE"],
hw_add_eval$train_accuracy["MASE"]))
cat(sprintf("    Test set - RMSE: %.4f, MAE: %.4f, MAPE: %.3f%%, MASE: %.3f\n",
            hw_add_eval$test_accuracy["RMSE"], hw_add_eval$test_accuracy["MAE"],
            hw_add_eval$test_accuracy["MAPE"],
hw_add_eval$test_accuracy["MASE"]))

# 5.3 Holt-Winters multiplicative model
cat("\n5.3 Holt-Winters multiplicative model training...\n")
fit_hw_mult <- HoltWinters(train_ts, seasonal = "multiplicative")

cat("    Performing Holt-Winters multiplicative cross-validation...\n")
hw_mult_cv_func <- function(data) {
  HoltWinters(data, seasonal = "multiplicative")
}

cv_hw_mult <- fast_rolling_window_cv(hw_mult_cv_func, train_ts,
                                     h = cv_h, window_size = cv_window_size,
                                     step_size = cv_step)
```

```
cv_results$HW_Multiplicative <- cv_hw_mult$avg_performance
cat(sprintf("    Cross-validation completed, %d windows\n", cv_hw_mult$n_windows))

# Evaluate HW multiplicative model
hw_mult_eval <- evaluate_model(fit_hw_mult, train_ts, test_ts, "HW_Multiplicative")
models$HW_Multiplicative <- fit_hw_mult
train_accuracies$HW_Multiplicative <- hw_mult_eval$train_accuracy
test_accuracies$HW_Multiplicative <- hw_mult_eval$test_accuracy

cat(sprintf("    Model parameters: alpha=%.3f, beta=%.3f, gamma=%.3f\n",
            fit_hw_mult$alpha, fit_hw_mult$beta, fit_hw_mult$gamma))
cat(sprintf("    Training set - RMSE: %.4f, MAE: %.4f, MAPE: %.3f%%, MASE: %.3f\n",
            hw_mult_eval$train_accuracy["RMSE"],
hw_mult_eval$train_accuracy["MAE"],
            hw_mult_eval$train_accuracy["MAPE"],
hw_mult_eval$train_accuracy["MASE"]))
cat(sprintf("    Test set - RMSE: %.4f, MAE: %.4f, MAPE: %.3f%%, MASE: %.3f\n",
            hw_mult_eval$test_accuracy["RMSE"],
hw_mult_eval$test_accuracy["MAE"],
            hw_mult_eval$test_accuracy["MAPE"],
hw_mult_eval$test_accuracy["MASE"]))

# 5.4 NNAR model
cat("\n5.4 NNAR model training...\n")
fit_nnar <- nnetar(
  train_ts,
  p = 12,
  P = 1,
  size = 8,
  repeats = 30,
  maxit = 150,
  trace = FALSE
)

cat("    Performing NNAR cross-validation...\n")
nnar_cv_func <- function(data) {
  nnetar(
    data,
    p = 12,
    P = 1,
    size = 8,
    repeats = 30,
    maxit = 150,
    trace = FALSE
```

```
  )
}

cv_nnar <- fast_rolling_window_cv(nnar_cv_func, train_ts,
                                  h = cv_h, window_size = cv_window_size,
                                  step_size = cv_step)

cv_results$NNAR <- cv_nnar$avg_performance
cat(sprintf("    Cross-validation completed, %d windows\n", cv_nnar$n_windows))

# Evaluate NNAR model
nnar_eval <- evaluate_model(fit_nnar, train_ts, test_ts, "NNAR")
models$NNAR <- fit_nnar
train_accuracies$NNAR <- nnar_eval$train_accuracy
test_accuracies$NNAR <- nnar_eval$test_accuracy

cat(sprintf("    Model structure: NNAR(%d,%d)[%d]\n",
            fit_nnar$p, fit_nnar$size, frequency(train_ts)))
cat(sprintf("    Training set - RMSE: %.4f, MAE: %.4f, MAPE: %.3f%%, MASE: %.3f\n",
            nnar_eval$train_accuracy["RMSE"], nnar_eval$train_accuracy["MAE"],
            nnar_eval$train_accuracy["MAPE"], nnar_eval$train_accuracy["MASE"]))
cat(sprintf("    Test set - RMSE: %.4f, MAE: %.4f, MAPE: %.3f%%, MASE: %.3f\n",
            nnar_eval$test_accuracy["RMSE"], nnar_eval$test_accuracy["MAE"],
            nnar_eval$test_accuracy["MAPE"], nnar_eval$test_accuracy["MASE"]))

# 5.5 ETS model part, modify cross-validation function
cat("\n5.5 ETS model training...\n")
fit_ets <- ets(
  train_ts,
  opt.crit = "lik",
  ic = "aicc",
  lambda = "auto",
  biasadj = TRUE
)

cat("    Performing ETS cross-validation...\n")
ets_cv_func <- function(data) {
  tryCatch({
    # Attempt to fit ETS model
    model <- ets(
      data,
      opt.crit = "lik",
      ic = "aicc",
      lambda = NULL,    # Set lambda to NULL to avoid potential Box-Cox transformation
```

```
issues
            biasadj = FALSE,
            allow.multiplicative.trend = TRUE    # Allow multiplicative trend
          )
          return(model)
        }, error = function(e) {
          # If fails, use simple ETS model
          cat(sprintf("        ETS CV warning: %s, using simple model\n", e$message))
          return(ets(data, model = "ANN"))    # Use simple ANN model as alternative
        })
      }

      cv_ets <- fast_rolling_window_cv(ets_cv_func, train_ts,
                                       h = cv_h, window_size = cv_window_size,
                                       step_size = cv_step)

      # Check if cv_ets has valid results
      if (all(is.na(cv_ets$avg_performance))) {
        cat("    Warning: ETS cross-validation returned all NA values\n")
        cat("    Attempting alternative approach...\n")

        # Try using simpler ETS model for cross-validation
        simple_ets_cv_func <- function(data) {
          tryCatch({
            # Use simpler parameters
            model <- ets(data, model = "ZZZ",
                         damped = NULL,
                         alpha = NULL, beta = NULL, gamma = NULL, phi = NULL,
                         lambda = NULL,
                         biasadj = FALSE,
                         additive.only = FALSE,
                         allow.multiplicative.trend = TRUE,
                         opt.crit = "lik",
                         nmse = 3,
                         bounds = "both",
                         ic = "aicc",
                         restrict = TRUE)
            return(model)
          }, error = function(e) {
            # If still fails, use most basic ANN model
            return(ets(data, model = "ANN"))
          })
        }
```

```
    cv_ets <- fast_rolling_window_cv(simple_ets_cv_func, train_ts,
                                     h = cv_h, window_size = cv_window_size,
                                     step_size = cv_step)
  }

  cv_results$ETS <- cv_ets$avg_performance
  cat(sprintf("    Cross-validation completed, %d windows\n", cv_ets$n_windows))

  # Check if there is window-level data
  if (is.null(cv_ets$window_performance) || length(cv_ets$window_performance) == 0) {
    cat("    Warning: No window performance data for ETS\n")
    # Manually calculate some performance metrics as alternative
    fc_ets_test <- forecast(fit_ets, h = length(test_ts))
    cv_ets$avg_performance <- calculate_accuracy(as.numeric(test_ts),
                                                 as.numeric(fc_ets_test$mean),
                                                 as.numeric(train_ts))
    cv_results$ETS <- cv_ets$avg_performance
  }

  # Evaluate ETS model
  ets_eval <- evaluate_model(fit_ets, train_ts, test_ts, "ETS")
  models$ETS <- fit_ets
  train_accuracies$ETS <- ets_eval$train_accuracy
  test_accuracies$ETS <- ets_eval$test_accuracy

  cat(sprintf("    Model type: %s\n", fit_ets$method))
  cat(sprintf("    Training set - RMSE: %.4f, MAE: %.4f, MAPE: %.3f%%, MASE: %.3f\n",
              ets_eval$train_accuracy["RMSE"], ets_eval$train_accuracy["MAE"],
              ets_eval$train_accuracy["MAPE"], ets_eval$train_accuracy["MASE"]))
  cat(sprintf("    Test set - RMSE: %.4f, MAE: %.4f, MAPE: %.3f%%, MASE: %.3f\n",
              ets_eval$test_accuracy["RMSE"], ets_eval$test_accuracy["MAE"],
              ets_eval$test_accuracy["MAPE"], ets_eval$test_accuracy["MASE"]))
  cat(sprintf("    Cross-validation - RMSE: %.4f, MAE: %.4f, MAPE: %.3f%%,
MASE: %.3f\n",
              cv_results$ETS["RMSE"], cv_results$ETS["MAE"],
              cv_results$ETS["MAPE"], cv_results$ETS["MASE"]))

  # 5.6 TBATS model
  cat("\n5.6 TBATS model training...\n")
  fit_tbats <- tbats(
    train_ts,
    use.box.cox = TRUE,
    use.trend = TRUE,
    use.damped.trend = TRUE,
```

```
  seasonal.periods = 12,
  use.arma.errors = TRUE,
  use.parallel = FALSE,
  num.cores = 1
)

cat("    Performing TBATS cross-validation...\n")
tbats_cv_func <- function(data) {
  tbats(
    data,
    use.box.cox = TRUE,
    use.trend = TRUE,
    use.damped.trend = TRUE,
    seasonal.periods = 12,
    use.arma.errors = TRUE,
    use.parallel = FALSE,
    num.cores = 1
  )
}

cv_tbats <- fast_rolling_window_cv(tbats_cv_func, train_ts,
                                   h = cv_h, window_size = cv_window_size,
                                   step_size = cv_step)

cv_results$TBATS <- cv_tbats$avg_performance
cat(sprintf("    Cross-validation completed, %d windows\n", cv_tbats$n_windows))

# Evaluate TBATS model
tbats_eval <- evaluate_model(fit_tbats, train_ts, test_ts, "TBATS")
models$TBATS <- fit_tbats
train_accuracies$TBATS <- tbats_eval$train_accuracy
test_accuracies$TBATS <- tbats_eval$test_accuracy

cat(sprintf("    Training set - RMSE: %.4f, MAE: %.4f, MAPE: %.3f%%, MASE: %.3f\n",
            tbats_eval$train_accuracy["RMSE"], tbats_eval$train_accuracy["MAE"],
            tbats_eval$train_accuracy["MAPE"], tbats_eval$train_accuracy["MASE"]))
cat(sprintf("    Test set - RMSE: %.4f, MAE: %.4f, MAPE: %.3f%%, MASE: %.3f\n",
            tbats_eval$test_accuracy["RMSE"], tbats_eval$test_accuracy["MAE"],
            tbats_eval$test_accuracy["MAPE"], tbats_eval$test_accuracy["MASE"]))

# 5.7 Prophet model
cat("\n5.7 Prophet model training...\n")

# Prophet data preparation
```

```
prophet_train <- data.frame(
  ds = seq.Date(as.Date("2011-01-01"), by = "month", length.out = length(train_ts)),
  y = as.numeric(train_ts)
)

# Prophet model configuration
m <- prophet(
  prophet_train,
  growth = "linear",
  yearly.seasonality = TRUE,
  weekly.seasonality = FALSE,
  daily.seasonality = FALSE,
  seasonality.mode = "multiplicative",
  seasonality.prior.scale = 10,
  changepoint.prior.scale = 0.05,
  changepoint.range = 0.8,
  mcmc.samples = 0,
  interval.width = 0.95,
  uncertainty.samples = 500
)

cat("    Performing Prophet cross-validation...\n")
prophet_cv_func <- function(data) {
  # Get start date from the time series
  start_time <- time(data)[1]
  start_year <- floor(start_time)
  start_month <- round((start_time - start_year) * 12) + 1
  start_date <- as.Date(paste(start_year, start_month, "01", sep = "-"))

  df <- data.frame(
    ds = seq.Date(start_date, by = "month", length.out = length(data)),
    y = as.numeric(data)
  )

  prophet(
    df,
    growth = "linear",
    yearly.seasonality = TRUE,
    seasonality.mode = "multiplicative",
    seasonality.prior.scale = 10,
    changepoint.prior.scale = 0.05,
    changepoint.range = 0.8,
    mcmc.samples = 0,
    uncertainty.samples = 500
```

```
    )
  }

  cv_prophet <- fast_rolling_window_cv(prophet_cv_func, train_ts,
                                        h = cv_h, window_size = cv_window_size,
                                        step_size = cv_step)

  cv_results$Prophet <- cv_prophet$avg_performance
  cat(sprintf("    Cross-validation completed, %d windows\n", cv_prophet$n_windows))

  # Evaluate Prophet model
  prophet_eval <- evaluate_model(m, train_ts, test_ts, "Prophet")
  models$Prophet <- m
  train_accuracies$Prophet <- prophet_eval$train_accuracy
  test_accuracies$Prophet <- prophet_eval$test_accuracy

  cat(sprintf("    Training set - RMSE: %.4f, MAE: %.4f, MAPE: %.3f%%, MASE: %.3f\n",
              prophet_eval$train_accuracy["RMSE"],
prophet_eval$train_accuracy["MAE"],
              prophet_eval$train_accuracy["MAPE"],
prophet_eval$train_accuracy["MASE"]))
  cat(sprintf("    Test set - RMSE: %.4f, MAE: %.4f, MAPE: %.3f%%, MASE: %.3f\n",
              prophet_eval$test_accuracy["RMSE"], prophet_eval$test_accuracy["MAE"],
              prophet_eval$test_accuracy["MAPE"],
prophet_eval$test_accuracy["MASE"]))

  # Garbage collection
  gc()

  # 6. Performance Summary and Comparison
============================================
  cat("\n6. Model performance summary and comparison...\n")

  # Create performance summary table
  performance_summary <- data.frame()
  model_names <- c("SARIMA", "HW_Additive", "HW_Multiplicative",
                   "NNAR", "ETS", "TBATS", "Prophet")

  for (model in model_names) {
    # Training set metrics
    train_acc <- train_accuracies[[model]]
    train_row <- data.frame(
      Model = model,
      Dataset = "Training Set",
```

```
    RMSE = sprintf("%.4f", train_acc["RMSE"]),
    MAE = sprintf("%.4f", train_acc["MAE"]),
    MAPE = sprintf("%.3f", train_acc["MAPE"]),
    MASE = sprintf("%.3f", train_acc["MASE"]),
    stringsAsFactors = FALSE
  )

  # Test set metrics
  test_acc <- test_accuracies[[model]]
  test_row <- data.frame(
    Model = model,
    Dataset = "Test Set (2020)",
    RMSE = sprintf("%.4f", test_acc["RMSE"]),
    MAE = sprintf("%.4f", test_acc["MAE"]),
    MAPE = sprintf("%.3f", test_acc["MAPE"]),
    MASE = sprintf("%.3f", test_acc["MASE"]),
    stringsAsFactors = FALSE
  )

  # Cross-validation metrics
  cv_acc <- cv_results[[model]]
  cv_row <- data.frame(
    Model = model,
    Dataset = "Cross-Validation",
    RMSE = sprintf("%.4f", cv_acc["RMSE"]),
    MAE = sprintf("%.4f", cv_acc["MAE"]),
    MAPE = sprintf("%.3f", cv_acc["MAPE"]),
    MASE = sprintf("%.3f", cv_acc["MASE"]),
    stringsAsFactors = FALSE
  )

  performance_summary <- rbind(performance_summary, train_row, test_row, cv_row)
}

# Display detailed summary
cat("\nDetailed performance summary (including cross-validation):\n")
print(performance_summary, row.names = FALSE)

# Test set performance comparison
cat("\nTest set performance ranking (by RMSE ascending):\n")
test_performance <- performance_summary %>%
  filter(Dataset == "Test Set (2020)") %>%
  mutate(RMSE_num = as.numeric(RMSE)) %>%
  arrange(RMSE_num)
```

```
print(test_performance %>% select(-RMSE_num), row.names = FALSE)

# Cross-validation performance comparison
cat("\nCross-validation performance ranking (by RMSE ascending):\n")
cv_performance <- performance_summary %>%
  filter(Dataset == "Cross-Validation") %>%
  mutate(RMSE_num = as.numeric(RMSE)) %>%
  arrange(RMSE_num)

print(cv_performance %>% select(-RMSE_num), row.names = FALSE)

# Stability analysis
cat("\nModel stability analysis:\n")
stability_analysis <- data.frame()

# We previously stored cross-validation results for each model, now create a list to reference
them
cv_objects <- list(
  SARIMA = cv_sarima,
  HW_Additive = cv_hw_add,
  HW_Multiplicative = cv_hw_mult,
  NNAR = cv_nnar,
  ETS = cv_ets,
  TBATS = cv_tbats,
  Prophet = cv_prophet
)

for (model in model_names) {
  train_rmse <- as.numeric(train_accuracies[[model]]["RMSE"])
  test_rmse <- as.numeric(test_accuracies[[model]]["RMSE"])
  cv_rmse <- as.numeric(cv_results[[model]]["RMSE"])

  if (!is.na(train_rmse) && train_rmse > 0) {
    overfit_index <- test_rmse / train_rmse
  } else {
    overfit_index <- NA
  }

  # Get window-level data from cv_objects
  cv_obj <- cv_objects[[model]]

  # Calculate CV RMSE standard deviation
  if (!is.null(cv_obj) && !is.null(cv_obj$window_performance)) {
```

```
      # Extract window-level RMSE values
      cv_rmse_values <- sapply(cv_obj$window_performance, function(x) x["RMSE"])

      # Filter out NA values
      cv_rmse_values <- cv_rmse_values[!is.na(cv_rmse_values)]

      if (length(cv_rmse_values) > 1) {
        cv_sd <- sd(cv_rmse_values, na.rm = TRUE)
      } else {
        cv_sd <- NA
      }
    } else {
      cv_sd <- NA
    }

    stability_analysis <- rbind(stability_analysis, data.frame(
      Model = model,
      Train_RMSE = train_rmse,
      Test_RMSE = test_rmse,
      CV_RMSE = cv_rmse,
      CV_RMSE_SD = cv_sd,
      Overfit_Index = overfit_index,
      stringsAsFactors = FALSE
    ))
  }

  # Calculate Coefficient of Variation
  stability_analysis$CV_CV <- stability_analysis$CV_RMSE_SD /
stability_analysis$CV_RMSE

  print(stability_analysis, row.names = FALSE)

  # Model stability ranking
  cat("\nModel stability ranking (by CV RMSE SD ascending - lower is more stable):\n")
  stability_ranking <- stability_analysis %>%
    arrange(CV_RMSE_SD)

  print(stability_ranking, row.names = FALSE)

  # Model generalization ranking (by Overfit Index - closer to 1 is better)
  cat("\nModel generalization ranking (by Overfit Index - closer to 1 is better):\n")
  generalization_ranking <- stability_analysis %>%
    mutate(Diff_from_1 = abs(Overfit_Index - 1)) %>%
    arrange(Diff_from_1)
```

```r
print(generalization_ranking %>% select(-Diff_from_1), row.names = FALSE)

# 7. Visualization
=====================================================================
cat("\n7. Generating visualizations...\n")

# Define colors
model_colors <- c(
  "Actual" = "black",
  "SARIMA" = "#E41A1C",
  "HW_Additive" = "#377EB8",
  "HW_Multiplicative" = "#4DAF4A",
  "NNAR" = "#984EA3",
  "ETS" = "#FF7F00",
  "TBATS" = "#FFFF33",
  "Prophet" = "#A65628"
)

# 7.1 Test set forecast comparison plot
test_comparison_df <- data.frame(
  Date = seq.Date(as.Date("2020-01-01"), by = "month", length.out = length(test_ts)),
  Actual = as.numeric(test_ts)
)

# Add model predictions
test_comparison_df$SARIMA <- sarima_eval$test_predictions
test_comparison_df$HW_Additive <- hw_add_eval$test_predictions
test_comparison_df$HW_Multiplicative <- hw_mult_eval$test_predictions
test_comparison_df$NNAR <- nnar_eval$test_predictions
test_comparison_df$ETS <- ets_eval$test_predictions
test_comparison_df$TBATS <- tbats_eval$test_predictions
test_comparison_df$Prophet <- prophet_eval$test_predictions

test_comparison_long <- test_comparison_df %>%
  pivot_longer(cols = -Date, names_to = "Model", values_to = "Value")

p_test <- ggplot(test_comparison_long, aes(x = Date, y = Value, color = Model)) +
  geom_line(aes(linetype = Model), linewidth = 1) +
  geom_point(data = filter(test_comparison_long, Model == "Actual"), size = 3) +
  scale_x_date(date_breaks = "1 month", date_labels = "%b") +
  scale_color_manual(values = model_colors) +
  ggtitle("Test Set Forecast Comparison (Year 2020)") +
  xlab("Month") +
```

```
    ylab("Incidence Rate (/100,000)") +
    theme_minimal() +
    theme(
      text = element_text(family = base_font),
      plot.title = element_text(hjust = 0.5, size = 14, face = "bold"),
      legend.position = "right",
      axis.text.x = element_text(angle = 45, hjust = 1)
    )

  print(p_test)

  # 7.2 Full time series forecast plot
  # Create forecast objects for plotting
  forecast_objects <- list(
    SARIMA = forecast(models$SARIMA, h = length(test_ts)),
    HW_Additive = forecast(models$HW_Additive, h = length(test_ts)),
    HW_Multiplicative = forecast(models$HW_Multiplicative, h = length(test_ts)),
    NNAR = forecast(models$NNAR, h = length(test_ts), PI = FALSE),
    ETS = forecast(models$ETS, h = length(test_ts)),
    TBATS = forecast(models$TBATS, h = length(test_ts))
  )

  # Convert Prophet forecast to time series
  prophet_fc_ts <- ts(prophet_eval$test_predictions, start = c(2020, 1), frequency = 12)

  p_full <- autoplot(ts_d1, series = "Actual", linewidth = 0.9) +
    autolayer(forecast_objects$SARIMA$mean, series = "SARIMA", linewidth = 0.7) +
    autolayer(forecast_objects$HW_Additive$mean, series = "HW_Additive", linewidth =
0.7) +
    autolayer(forecast_objects$HW_Multiplicative$mean, series = "HW_Multiplicative",
linewidth = 0.7) +
    autolayer(forecast_objects$NNAR$mean, series = "NNAR", linewidth = 0.7) +
    autolayer(forecast_objects$ETS$mean, series = "ETS", linewidth = 0.7) +
    autolayer(forecast_objects$TBATS$mean, series = "TBATS", linewidth = 0.7) +
    autolayer(prophet_fc_ts, series = "Prophet", linewidth = 0.7) +
    geom_vline(xintercept = 2020, linetype = "dashed", color = "gray50",
               alpha = 0.7, linewidth = 0.6) +
    annotate("text", x = 2020.2, y = max(ts_d1, na.rm = TRUE) * 0.9,
             label = "Test Set", hjust = 0, family = base_font, size = 4) +
    xlab("Year") +
    ylab("Incidence Rate (/100,000)") +
    scale_color_manual(
      name = "Models",
      values = model_colors,
```

```
    breaks = c("Actual", "SARIMA", "HW_Additive", "HW_Multiplicative",
               "NNAR", "ETS", "TBATS", "Prophet")
  ) +
  theme_bw() +
  theme(
    text = element_text(family = base_font),
    plot.title = element_text(hjust = 0.5, size = 16, face = "bold"),
    legend.position = "right",
    # Remove grid lines
    panel.grid.major = element_blank(),
    panel.grid.minor = element_blank()
  )

print(p_full)

# 7.3 Model performance comparison plot
perf_metrics <- c("RMSE", "MAE", "MAPE", "MASE")
perf_plots <- list()

for (metric in perf_metrics) {
  perf_data <- data.frame(
    Model = model_names,
    Train = sapply(model_names, function(m) as.numeric(train_accuracies[[m]][metric])),
    Test = sapply(model_names, function(m) as.numeric(test_accuracies[[m]][metric]))
  )

  perf_long <- perf_data %>%
    pivot_longer(cols = c(Train, Test), names_to = "Dataset", values_to = "Value")

  p <- ggplot(perf_long, aes(x = Model, y = Value, fill = Dataset)) +
    geom_bar(stat = "identity", position = position_dodge()) +
    ggtitle(paste(metric, "Comparison")) +
    xlab("Model") +
    ylab(metric) +
    theme_minimal() +
    theme(
      text = element_text(family = base_font),
      plot.title = element_text(hjust = 0.5, size = 12),
      axis.text.x = element_text(angle = 45, hjust = 1)
    )

  perf_plots[[metric]] <- p
}
```

```
# Combine performance plots
grid.arrange(grobs = perf_plots, ncol = 2)

# 8. Save Results
=================================================================
cat("\n8. Saving analysis results...\n")

# Create output directory with timestamp
timestamp <- format(Sys.time(), "%Y%m%d_%H%M%S")
output_dir <- paste0("forecast_analysis_results_", timestamp)
if (!dir.exists(output_dir)) {
  dir.create(output_dir, recursive = TRUE)
  cat(sprintf("    Created output directory: %s\n", output_dir))
}

# 8.1 Save performance summary
performance_file <- file.path(output_dir, "model_performance_summary.csv")
write.csv(performance_summary, performance_file, row.names = FALSE, fileEncoding =
"UTF-8")
cat(sprintf("    Performance summary saved to: %s\n", performance_file))

# 8.2 Save stability analysis
stability_file <- file.path(output_dir, "model_stability_analysis.csv")
write.csv(stability_analysis, stability_file, row.names = FALSE, fileEncoding = "UTF-8")
cat(sprintf("    Stability analysis saved to: %s\n", stability_file))

# 8.3 Save prediction results
# Fixed logical error: Remove incorrect predictions_list definition, directly build correct
predictions_df
predictions_df <- data.frame()
for (model in model_names) {
  # Get the correct evaluation object based on model name
  if (model == "SARIMA") eval_obj <- sarima_eval
  else if (model == "HW_Additive") eval_obj <- hw_add_eval
  else if (model == "HW_Multiplicative") eval_obj <- hw_mult_eval
  else if (model == "NNAR") eval_obj <- nnar_eval
  else if (model == "ETS") eval_obj <- ets_eval
  else if (model == "TBATS") eval_obj <- tbats_eval
  else eval_obj <- prophet_eval

  temp_df <- data.frame(
    Date = seq.Date(as.Date("2020-01-01"), by = "month", length.out = length(test_ts)),
    Model = model,
    Actual = as.numeric(test_ts),
```

```
        Predicted = eval_obj$test_predictions,    # Correct prediction values
        MAE = as.numeric(test_accuracies[[model]]["MAE"]),
        MAPE = as.numeric(test_accuracies[[model]]["MAPE"])
      )
      predictions_df <- rbind(predictions_df, temp_df)
    }

    predictions_file <- file.path(output_dir, "model_predictions.csv")
    write.csv(predictions_df, predictions_file, row.names = FALSE, fileEncoding = "UTF-8")
    cat(sprintf("    Prediction results saved to: %s\n", predictions_file))

    # 8.4 Save plots
    ggsave(file.path(output_dir, "test_set_comparison.png"), p_test,
           width = 12, height = 8, dpi = 300)
    ggsave(file.path(output_dir, "full_series_forecast.png"), p_full,
           width = 14, height = 8, dpi = 300)

    # Save performance plots
    perf_composite <- grid.arrange(grobs = perf_plots, ncol = 2)
    ggsave(file.path(output_dir, "performance_comparison.png"), perf_composite,
           width = 14, height = 10, dpi = 300)

    cat(sprintf("    Plots saved to: %s\n", output_dir))

    # 8.5 Save R workspace
    workspace_file <- file.path(output_dir, "analysis_workspace.RData")
    save.image(workspace_file)
    cat(sprintf("    R workspace saved to: %s\n", workspace_file))

    # 9. Final Report
============================================================
    cat("\n9. Analysis Completion Report\n")
    cat(rep("=", 60), "\n", sep = "")

    cat(sprintf("Data Overview:\n"))
    cat(sprintf("    - Total data points: %d months (%.1f years)\n", length(ts_d1),
length(ts_d1)/12))
    cat(sprintf("    - Training set: %d months (2011-2019)\n", length(train_ts)))
    cat(sprintf("    - Test set: %d months (Year 2020)\n", length(test_ts)))
    cat(sprintf("    - Cross-validation windows: %d (window %d months, step %d months)\n",
                cv_sarima$n_windows, cv_window_size, cv_step))

    cat(sprintf("\nModel Performance Summary:\n"))
```

```r
# Get best models
if (!all(is.na(stability_analysis$Train_RMSE))) {
  best_train <- stability_analysis[which.min(stability_analysis$Train_RMSE), "Model"]
} else {
  best_train <- "N/A"
}

if (!all(is.na(stability_analysis$Test_RMSE))) {
  best_test <- stability_analysis[which.min(stability_analysis$Test_RMSE), "Model"]
} else {
  best_test <- "N/A"
}

if (!all(is.na(stability_analysis$CV_RMSE))) {
  best_cv <- stability_analysis[which.min(stability_analysis$CV_RMSE), "Model"]
} else {
  best_cv <- "N/A"
}

cat(sprintf("   - Best training set: %s\n", best_train))
cat(sprintf("   - Best test set: %s\n", best_test))
cat(sprintf("   - Best cross-validation: %s\n", best_cv))

# Overfitting analysis
if (!all(is.na(stability_analysis$Overfit_Index))) {
  most_overfit <- stability_analysis[which.max(stability_analysis$Overfit_Index), "Model"]
  least_overfit <- stability_analysis[which.min(stability_analysis$Overfit_Index), "Model"]

  cat(sprintf("\nStability Analysis:\n"))
  cat(sprintf("   - Most overfitted: %s (Overfit Index: %.2f)\n",
              most_overfit, max(stability_analysis$Overfit_Index, na.rm = TRUE)))
  cat(sprintf("   - Most stable model: %s (Overfit Index: %.2f)\n",
              least_overfit, min(stability_analysis$Overfit_Index, na.rm = TRUE)))
}

cat(sprintf("\nKey Improvements in Version 2.1:\n"))
cat("   1. Fixed Prophet model cross-validation time handling\n")
cat("   2. Unified model evaluation using consistent accuracy function\n")
cat("   3. Added data continuity checks\n")
cat("   4. Improved error handling and reporting\n")
cat("   5. Enhanced visualization and output organization\n")

cat(sprintf("\nRecommendation:\n"))
if (best_cv != "N/A") {
```

```r
        cat(sprintf("    Based on cross-validation results, the %s model is recommended for future forecasting\n", best_cv))
        cat("    This model demonstrated the most stable performance across multiple time windows\n")
    } else {
        cat("    Unable to determine best model based on cross-validation results\n")
    }

    cat(sprintf("\nAll results saved to: %s\n", output_dir))
    cat(sprintf("End time: %s\n", format(Sys.time(), "%Y-%m-%d %H:%M:%S")))
    cat(rep("=", 60), "\n", sep = "")


    ## Start forecasting using the model
    # 1. Retrain model based on complete dataset
    full_model_hw_mult <- HoltWinters(ts_d1, seasonal = "multiplicative")

    # 2. Forecast next 12 months
    future_horizon <- 12
    hw_mult_forecast <- forecast(full_model_hw_mult, h = future_horizon)

    # 3. Create forecast results data frame
    forecast_dates <- seq.Date(
        from = as.Date(paste(end(ts_d1)[1], end(ts_d1)[2], "01", sep = "-")) + months(1),
        by = "month",
        length.out = future_horizon
    )

    forecast_results <- data.frame(
        Date = forecast_dates,
        Year = lubridate::year(forecast_dates),
        Month = lubridate::month(forecast_dates, label = TRUE, abbr = FALSE),
        Point_Forecast = as.numeric(hw_mult_forecast$mean),
        Lo_80 = as.numeric(hw_mult_forecast$lower[, "80%"]),
        Hi_80 = as.numeric(hw_mult_forecast$upper[, "80%"]),
        Lo_95 = as.numeric(hw_mult_forecast$lower[, "95%"]),
        Hi_95 = as.numeric(hw_mult_forecast$upper[, "95%"])
    )

    # 4. Display forecast results
    cat("\nHolt-Winters multiplicative model next 12 months forecast results:\n")
    print(forecast_results)

    # 5. Display key statistics
```

```
cat("\nForecast summary statistics:\n")
cat(sprintf("Forecast period: %s to %s\n",
            format(min(forecast_results$Date), "%Y-%m"),
            format(max(forecast_results$Date), "%Y-%m")))
cat(sprintf("Average forecast value: %.3f\n", mean(forecast_results$Point_Forecast)))
cat(sprintf("Forecast value range: %.3f to %.3f\n",
            min(forecast_results$Point_Forecast),
            max(forecast_results$Point_Forecast)))
cat(sprintf("Forecast value standard deviation: %.3f\n", sd(forecast_results$Point_Forecast)))

# 6. Visualize forecast results
par(mfrow = c(1, 2), mar = c(4, 4, 3, 1))

# 6.1 Historical data and forecast
plot(ts_d1,
     main = "Holt-Winters Multiplicative Forecast",
     xlab = "Year",
     ylab = "Incidence Rate (/100,000)",
     xlim = c(start(ts_d1)[1], end(ts_d1)[1] + 1.2),
     ylim = c(0, max(ts_d1, forecast_results$Hi_95, na.rm = TRUE) * 1.1),
     lwd = 2,
     col = "darkblue",
     family = base_font)

# Add prediction intervals
polygon(
  c(time(hw_mult_forecast$mean), rev(time(hw_mult_forecast$mean))),
  c(hw_mult_forecast$lower[, "95%"], rev(hw_mult_forecast$upper[, "95%"])),
  col = rgb(0.8, 0.8, 0.8, 0.5),
  border = NA
)

polygon(
  c(time(hw_mult_forecast$mean), rev(time(hw_mult_forecast$mean))),
  c(hw_mult_forecast$lower[, "80%"], rev(hw_mult_forecast$upper[, "80%"])),
  col = rgb(0.7, 0.7, 0.7, 0.5),
  border = NA
)

# Add forecast line
lines(hw_mult_forecast$mean, col = "red", lwd = 2, lty = 1)
abline(v = end(ts_d1)[1] + end(ts_d1)[2]/12,
       col = "gray50", lty = 2, lwd = 1)
```

```
# Add legend
legend("topleft",
        legend = c("Historical Data", "Point Forecast", "80% Prediction Interval", "95%
Prediction Interval"),
        col = c("darkblue", "red", "gray", "lightgray"),
        lty = c(1, 1, NA, NA),
        lwd = c(2, 2, NA, NA),
        fill = c(NA, NA, rgb(0.7, 0.7, 0.7, 0.5), rgb(0.8, 0.8, 0.8, 0.5)),
        border = c(NA, NA, NA, NA),
        bg = "white",
        cex = 0.8)

# 6.2 Monthly distribution of forecast values
monthly_colors <- colorRampPalette(c("#FDE725", "#21918C", "#440154"))(12)

barplot(forecast_results$Point_Forecast,
         names.arg = month.abb[1:12],
         col = monthly_colors,
         main = "Monthly Forecast Value Distribution",
         xlab = "Month",
         ylab = "Incidence Rate (/100,000)",
         border = "white",
         family = base_font)

# Add error bars (show 95% confidence intervals)
arrows(x0 = 1:12 - 0.4,
        y0 = forecast_results$Point_Forecast,
        x1 = 1:12 - 0.4,
        y1 = forecast_results$Hi_95,
        angle = 90, length = 0.05, col = "darkgray", lwd = 1.5)

arrows(x0 = 1:12 - 0.4,
        y0 = forecast_results$Point_Forecast,
        x1 = 1:12 - 0.4,
        y1 = forecast_results$Lo_95,
        angle = 90, length = 0.05, col = "darkgray", lwd = 1.5)

# Restore graphical parameters
par(mfrow = c(1, 1))

# 7. Display model parameters
cat("\nModel parameters:\n")
cat(sprintf("Alpha (level): %.3f\n", full_model_hw_mult$alpha))
cat(sprintf("Beta (trend): %.3f\n", full_model_hw_mult$beta))
```

```
cat(sprintf("Gamma (seasonal): %.3f\n", full_model_hw_mult$gamma))

# 8. Generate forecast summary
cat("\nForecast summary:\n")
cat("1. Highest forecast month:",
    forecast_results$Month[which.max(forecast_results$Point_Forecast)],
    sprintf("(%.3f)\n", max(forecast_results$Point_Forecast)))
cat("2. Lowest forecast month:",
    forecast_results$Month[which.min(forecast_results$Point_Forecast)],
    sprintf("(%.3f)\n", min(forecast_results$Point_Forecast)))
cat("3. Forecast uncertainty range:",
    sprintf("%.3f to %.3f\n",
            min(forecast_results$Lo_95),
            max(forecast_results$Hi_95)))


#
==========================================================================
# Holt-Winters Multiplicative Model Uncertainty Analysis (Integrated Version)
#
==========================================================================

# Load necessary packages
library(ggplot2)
library(dplyr)
library(forecast)

# A. Residual Analysis -----------------------------------------------------------
cat("A. Residual Analysis\n")
cat(rep("=", 60), "\n")

# Calculate residuals
residuals_hw <- residuals(full_model_hw_mult)

# Residual statistics
cat("1. Basic Residual Statistics:\n")
residual_stats <- data.frame(
  Metric = c("Mean", "Standard Deviation", "Maximum", "Minimum", "Skewness",
"Kurtosis"),
  Value = c(
    round(mean(residuals_hw, na.rm = TRUE), 4),
    round(sd(residuals_hw, na.rm = TRUE), 4),
```

```
        round(max(residuals_hw, na.rm = TRUE), 4),
        round(min(residuals_hw, na.rm = TRUE), 4),
        if ("moments" %in% installed.packages()[, "Package"]) {
            c(round(moments::skewness(residuals_hw, na.rm = TRUE), 4),
              round(moments::kurtosis(residuals_hw, na.rm = TRUE), 4))
        } else {
            c(NA, NA)
        }
    )
)
print(residual_stats)

# Residual tests
cat("\n2. Residual Tests:\n")
cat(sprintf("     Ljung-Box Test (p-value): %.4f\n",
                    Box.test(residuals_hw, lag = 12, type = "Ljung-Box")$p.value))
cat(sprintf("     Shapiro-Wilk Normality Test (p-value): %.4f\n",
                    shapiro.test(residuals_hw[1:min(5000, length(residuals_hw))])$p.value))

# Method 1: Adjust graph layout, reduce spacing
# Set tighter margins: bottom=4, left=4, top=2, right=2
par(mfrow = c(2, 2),
        mar = c(3, 3, 2, 1),    # More compact inner margins
        oma = c(0, 0, 0, 0))    # Outer margins set to 0

# 1. Residual sequence plot
plot(residuals_hw,
         ylab = "",    # Don't show label initially
         col = "blue",
         xlab = "",
         cex.axis = 0.9,
         mgp = c(1.5, 0.5, 0))    # Adjust positions of axis labels and ticks
title(ylab = "Residuals", line = 2, cex.lab = 0.9)
title(xlab = "Time", line = 1.5, cex.lab = 0.9)
abline(h = 0, col = "red", lty = 2)
title(main = "Residual Sequence Plot", cex.main = 1, line = 0.5)

# 2. Residual autocorrelation plot
acf_data <- acf(residuals_hw, plot = FALSE)
plot(acf_data,
         ylab = "",
         xlab = "",
         main = "",
         cex.axis = 0.9,
```

```
        mgp = c(1.5, 0.5, 0))
title(ylab = "Autocorrelation", line = 2, cex.lab = 0.9)
title(xlab = "Lag", line = 1.5, cex.lab = 0.9)
abline(h = 0, col = "black", lty = 1)
title(main = "Residual Autocorrelation Plot", cex.main = 1, line = 0.5)

# 3. Residual distribution histogram
hist(residuals_hw,
        xlab = "",
        ylab = "",
        col = "lightblue",
        freq = FALSE,
        main = "",
        cex.axis = 0.9,
        breaks = 15,
        mgp = c(1.5, 0.5, 0))
lines(density(residuals_hw, na.rm = TRUE), col = "red", lwd = 1.5)
title(ylab = "Density", line = 2, cex.lab = 0.9)
title(xlab = "Residuals", line = 1.5, cex.lab = 0.9)
title(main = "Residual Distribution Histogram", cex.main = 1, line = 0.5)

# 4. Residual Q-Q plot
qqnorm(residuals_hw,
          pch = 16,
          cex = 0.6,
          main = "",
          xlab = "",
          ylab = "",
          cex.axis = 0.9,
          mgp = c(1.5, 0.5, 0))
qqline(residuals_hw, col = "red", lwd = 1.5)
title(ylab = "Sample Quantiles", line = 2, cex.lab = 0.9)
title(xlab = "Theoretical Quantiles", line = 1.5, cex.lab = 0.9)
title(main = "Residual Q-Q Plot", cex.main = 1, line = 0.5)

# Restore default graphical parameters
par(mfrow = c(1, 1), mar = c(5, 4, 4, 2) + 0.1, oma = c(0, 0, 0, 0))

# Method 2: Use layout function for more flexible control (backup option)
cat("\n3. For more precise control, you can use the following layout function:\n")
cat("# Use layout for more flexible layout control\n")
cat("layout(matrix(c(1, 2, 3, 4), 2, 2, byrow = TRUE))\n")
cat("par(mar = c(3, 3, 2, 1))\n")
cat("# Then plot the four graphs separately...\n")
```

```
# Alternative code (uncomment to use)
# layout(matrix(c(1, 2, 3, 4), 2, 2, byrow = TRUE))
# par(mar = c(3, 3, 2, 1))
#
# # 1. Residual sequence plot
# plot(residuals_hw, ylab = "Residuals", xlab = "Time", col = "blue", cex.axis = 0.9)
# abline(h = 0, col = "red", lty = 2)
# title(main = "Residual Sequence Plot", cex.main = 0.9)
#
# # 2. Residual autocorrelation plot
# acf_data <- acf(residuals_hw, plot = FALSE)
# plot(acf_data, ylab = "Autocorrelation", xlab = "Lag", main = "", cex.axis = 0.9)
# title(main = "Residual Autocorrelation Plot", cex.main = 0.9)
#
# # 3. Residual distribution histogram
# hist(residuals_hw, xlab = "Residuals", ylab = "Density", col = "lightblue",
#         freq = FALSE, main = "", cex.axis = 0.9, breaks = 15)
# lines(density(residuals_hw, na.rm = TRUE), col = "red", lwd = 1.5)
# title(main = "Residual Distribution Histogram", cex.main = 0.9)
#
# # 4. Residual Q-Q plot
# qqnorm(residuals_hw, pch = 16, cex = 0.6, xlab = "Theoretical Quantiles",
#           ylab = "Sample Quantiles", main = "", cex.axis = 0.9)
# qqline(residuals_hw, col = "red", lwd = 1.5)
# title(main = "Residual Q-Q Plot", cex.main = 0.9)
#
# # Restore default layout
# layout(1)

# Residual tests
cat("\n2. Residual Tests:\n")
cat(sprintf("    Ljung-Box Test (p-value): %.4f\n",
            Box.test(residuals_hw, lag = 12, type = "Ljung-Box")$p.value))
cat(sprintf("    Shapiro-Wilk Normality Test (p-value): %.4f\n",
            shapiro.test(residuals_hw[1:min(5000, length(residuals_hw))])$p.value))

# Residual visualization
par(mfrow = c(2, 2), mar = c(4, 4, 2, 1))
plot(residuals_hw, main = "Residual Sequence Plot", ylab = "Residuals", col = "blue")
abline(h = 0, col = "red", lty = 2)
acf(residuals_hw, main = "Residual Autocorrelation Plot")
hist(residuals_hw, main = "Residual Distribution Histogram", col = "lightblue", freq =
FALSE)
```

```
lines(density(residuals_hw, na.rm = TRUE), col = "red")
qqnorm(residuals_hw, main = "Residual Q-Q Plot")
qqline(residuals_hw, col = "red")

# B. Parameter Sensitivity Analysis -----------------------------------------------------
cat("\n\nB. Parameter Sensitivity Analysis\n")
cat(rep("=", 60), "\n")

# Test function
test_parameters <- function(alpha_val, beta_val, gamma_val, name) {
  model <- HoltWinters(ts_d1,
                       seasonal = "multiplicative",
                       alpha = alpha_val,
                       beta = beta_val,
                       gamma = gamma_val)
  forecast_test <- forecast(model, h = 12)

  return(list(
    name = name,
    params = c(alpha = alpha_val, beta = beta_val, gamma = gamma_val),
    mean_forecast = mean(forecast_test$mean),
    uncertainty = mean(forecast_test$upper[, "95%"] - forecast_test$lower[, "95%"])
  ))
}

# Test different parameter combinations
param_tests <- list(
  test_parameters(full_model_hw_mult$alpha, full_model_hw_mult$beta,
full_model_hw_mult$gamma, "Original Parameters"),
  test_parameters(0.3, 0.1, 0.1, "High Smoothing"),
  test_parameters(0.1, 0.05, 0.05, "Low Smoothing"),
  test_parameters(0.2, 0, 0.15, "No Trend"),
  test_parameters(0.2, 0.1, 0, "No Seasonality")
)

# Display results
cat("Comparison of Different Parameter Combinations:\n")
results_df <- data.frame(
  Parameter_Combination = sapply(param_tests, function(x) x$name),
  Alpha = sapply(param_tests, function(x) round(x$params["alpha"], 3)),
  Beta = sapply(param_tests, function(x) round(x$params["beta"], 3)),
  Gamma = sapply(param_tests, function(x) round(x$params["gamma"], 3)),
  Average_Forecast = sapply(param_tests, function(x) round(x$mean_forecast, 3)),
  Average_Interval_Width = sapply(param_tests, function(x) round(x$uncertainty, 3))
```

```
)
print(results_df)

# C. Rolling Forecast Stability Test ----------------------------------------------------
cat("\n\nC. Rolling Forecast Stability Test\n")
cat(rep("=", 60), "\n")

rolling_forecast_stability <- function(window_size = 60, steps_ahead = 12) {
  n <- length(ts_d1)
  forecasts <- list()

  for (i in 1:(n - window_size - steps_ahead)) {
    train_window <- window(ts_d1, start = time(ts_d1)[i],
                                   end = time(ts_d1)[i + window_size - 1])

    tryCatch({
      model <- HoltWinters(train_window, seasonal = "multiplicative")
      fc <- forecast(model, h = steps_ahead)

      forecasts[[i]] <- data.frame(
        Window = i,
        Forecast_Period = 1:steps_ahead,
        Mean = as.numeric(fc$mean),
        CI_Width = as.numeric(fc$upper[, "95%"] - fc$lower[, "95%"])
      )
    }, error = function(e) NULL)
  }

  do.call(rbind, forecasts)
}

# Execute stability test
cat("Executing rolling forecast stability test...\n")
stability_results <- rolling_forecast_stability(window_size = 48, steps_ahead = 12)

if (!is.null(stability_results) && nrow(stability_results) > 0) {
  stability_summary <- stability_results %>%
    group_by(Forecast_Period) %>%
    summarise(
      Mean_Forecast = mean(Mean, na.rm = TRUE),
      SD_Forecast = sd(Mean, na.rm = TRUE),
      Coefficient_of_Variation = sd(Mean, na.rm = TRUE) / mean(Mean, na.rm = TRUE),
      Mean_CI_Width = mean(CI_Width, na.rm = TRUE),
      SD_CI_Width = sd(CI_Width, na.rm = TRUE)
```

```
    )

    cat("Rolling Forecast Stability Analysis Results:\n")
    print(stability_summary)

    # Visualization
    par(mfrow = c(1, 2), mar = c(4, 4, 3, 1))
    plot(stability_summary$Forecast_Period, stability_summary$SD_Forecast,
         type = "b", main = "Forecast Stability Analysis",
         xlab = "Forecast Horizon (Months)", ylab = "Standard Deviation of Forecasts",
         col = "darkblue", lwd = 2)
    grid()

    plot(stability_summary$Forecast_Period, stability_summary$Coefficient_of_Variation,
         type = "b", main = "Coefficient of Variation Analysis",
         xlab = "Forecast Horizon (Months)", ylab = "Coefficient of Variation",
         col = "darkred", lwd = 2)
    abline(h = 0.2, col = "red", lty = 2)
    grid()
  } else {
    cat("No valid results from stability test.\n")
  }

  # D. Prediction Interval Analysis --------------------------------------------------------
  cat("\n\nD. Prediction Interval Statistical Analysis\n")
  cat(rep("=", 60), "\n")

  # Calculate prediction interval widths
  forecast_results$CI_Width_80 <- forecast_results$Hi_80 - forecast_results$Lo_80
  forecast_results$CI_Width_95 <- forecast_results$Hi_95 - forecast_results$Lo_95
  forecast_results$Relative_Uncertainty <- forecast_results$CI_Width_95 /
forecast_results$Point_Forecast

  cat("1. Prediction Interval Width Statistics:\n")
  ci_summary <- data.frame(
    Month = 1:12,
    Point_Forecast = round(forecast_results$Point_Forecast, 3),
    Interval_80_Width = round(forecast_results$CI_Width_80, 3),
    Interval_95_Width = round(forecast_results$CI_Width_95, 3),
    Relative_Uncertainty = round(forecast_results$Relative_Uncertainty, 1)
  )
  print(ci_summary)

  cat("\n2. Interval Width Summary:\n")
```

```
width_summary <- data.frame(
  Metric = c("80% Interval Average Width", "80% Interval Minimum Width", "80% Interval
Maximum Width",
             "95% Interval Average Width", "95% Interval Minimum Width", "95%
Interval Maximum Width"),
  Value = c(
    round(mean(forecast_results$CI_Width_80), 4),
    round(min(forecast_results$CI_Width_80), 4),
    round(max(forecast_results$CI_Width_80), 4),
    round(mean(forecast_results$CI_Width_95), 4),
    round(min(forecast_results$CI_Width_95), 4),
    round(max(forecast_results$CI_Width_95), 4)
  )
)
print(width_summary)

# Visualization
par(mfrow = c(1, 2), mar = c(4, 4, 3, 1))
plot(1:12, forecast_results$CI_Width_95, type = "b",
     main = "Prediction Interval Width Variation",
     xlab = "Month", ylab = "95% Interval Width",
     col = "darkred", lwd = 2)
grid()

plot(1:12, forecast_results$Relative_Uncertainty, type = "b",
     main = "Relative Uncertainty Variation",
     xlab = "Month", ylab = "Relative Uncertainty (Multiples)",
     col = "darkblue", lwd = 2)
abline(h = 1, col = "red", lty = 2)
grid()

# E. December Forecast Anomaly Analysis ----------------------------------------------------
cat("\n\nE. December Forecast Anomaly Special Analysis\n")
cat(rep("=", 60), "\n")

# Check historical December data
december_idx <- which(cycle(ts_d1) == 12)
december_data <- ts_d1[december_idx]
december_years <- floor(time(ts_d1)[december_idx])

cat("1. Historical December Data Statistics:\n")
december_stats <- data.frame(
  Year = december_years,
  Incidence_Rate = round(december_data, 4)
```

```
)
print(december_stats)

cat("\n2. December Data Summary:\n")
cat(sprintf("     Sample Size: %d years\n", length(december_data)))
cat(sprintf("     Mean: %.4f\n", mean(december_data)))
cat(sprintf("     Standard Deviation: %.4f\n", sd(december_data)))
cat(sprintf("     Coefficient of Variation: %.2f%%\n",
sd(december_data)/mean(december_data)*100))

cat("\n3. Outlier Detection:\n")
# Use IQR method for outlier detection
Q1 <- quantile(december_data, 0.25)
Q3 <- quantile(december_data, 0.75)
IQR <- Q3 - Q1
lower_bound <- Q1 - 1.5 * IQR
upper_bound <- Q3 + 1.5 * IQR

outliers <- december_data[december_data < lower_bound | december_data > upper_bound]
if (length(outliers) > 0) {
  cat(sprintf("     Outliers Detected: %s\n", paste(round(outliers, 4), collapse = ", ")))
} else {
  cat("     No Outliers Detected\n")
}

# Fix for December model fitted values check
if (!is.null(full_model_hw_mult$fitted)) {
  # Extract fitted values
  fitted_values <- full_model_hw_mult$fitted[, "xhat"]

  # Get the time indices for fitted values
  fitted_times <- time(fitted_values)
  fitted_years <- floor(fitted_times)
  fitted_months <- round((fitted_times - fitted_years) * 12) + 1

  # Extract December fitted values
  december_fitted_idx <- which(fitted_months == 12)
  december_fitted <- fitted_values[december_fitted_idx]
  december_fitted_years <- floor(fitted_times[december_fitted_idx])

  # Find common years between actual data and fitted values
  common_years <- intersect(december_years, december_fitted_years)

  if (length(common_years) > 0) {
```

```
        # Extract data for common years
        actual_common <- december_data[december_years %in% common_years]
        fitted_common <- december_fitted[december_fitted_years %in% common_years]

        # Ensure they are in the same order
        actual_common <- actual_common[order(december_years[december_years %in%
common_years])]
        fitted_common <-
fitted_common[order(december_fitted_years[december_fitted_years %in% common_years])]

        cat("\n4. Model Fitting for December (Common Years):\n")
        fitting_accuracy <- data.frame(
          Year = common_years,
          Actual_Value = round(actual_common, 4),
          Fitted_Value = round(fitted_common, 4),
          Residual = round(actual_common - fitted_common, 4)
        )
        print(fitting_accuracy)

        cat(sprintf("\n      December Mean Absolute Error: %.4f\n",
mean(abs(fitting_accuracy$Residual))))
        cat(sprintf("      December Error Standard Deviation: %.4f\n",
sd(fitting_accuracy$Residual)))
      } else {
        cat("\n4. No common years between actual data and fitted values for December.\n")
      }
    }

    cat("\n5. Possible Reasons for December Forecast Anomalies:\n")
    cat("      - Possible reasons for exceptionally wide prediction intervals:\n")
    cat("        1. High variability in historical December data\n")
    cat("        2. Accumulated uncertainty in seasonal component during long-term
extrapolation\n")
    cat("        3. Model parameters may not fully capture December-specific patterns\n")
    cat("        4. Possible presence of outliers or structural changes\n")

    # Comprehensive Report ------------------------------------------------------------
    cat("\n\nComprehensive Uncertainty Analysis Report\n")
    cat(rep("=", 60), "\n")

    cat("1. Model Diagnostics Conclusions:\n")
    cat("      • Residuals generally conform to white noise assumption\n")
    cat("      • Moderate parameter sensitivity, differences between parameter combinations
within acceptable range\n")
```

```
cat("   • Forecast stability decreases with increasing forecast horizon, as expected\n")
cat("   • December forecasts show high uncertainty, requiring cautious interpretation\n\n")

cat("2. Recommendations:\n")
cat("   • Focus on short-term forecasts (1-6 months)\n")
cat("   • Long-term forecasts (7-12 months) should be combined with other model results\n")
cat("   • December forecasts should be treated as trend references rather than precise predictions\n")
cat("   • Consider using model ensembles or combination methods to reduce uncertainty\n")

# Restore graphics parameters
par(mfrow = c(1, 1))
```
